# Northern protected areas will become important refuges for biodiversity tracking suitable climates

**DOI:** 10.1038/s41598-018-23050-w

**Published:** 2018-03-15

**Authors:** Dominique Berteaux, Marylène Ricard, Martin-Hugues St-Laurent, Nicolas Casajus, Catherine Périé, Frieda Beauregard, Sylvie de Blois

**Affiliations:** 1 Canada Research Chair on Northern Biodiversity, Centre for Northern Studies and Quebec Centre for Biodiversity Science, Université du Québec à Rimouski, 300 allée des Ursulines, Rimouski, QC G5L 3A1 Canada; 2 Centre for Northern Studies, Centre for Forest Research, Université du Québec à Rimouski, 300 allée des Ursulines, Rimouski, QC G5L 3A1 Canada; 3 Direction de la recherche forestière, Ministère des Forêts, de la Faune et des Parcs, 2700, rue Einstein, C.1.200, Québec, QC G1P 3W8 Canada; 4 Department of Plant Science, Macdonald Campus, McGill University, 21111 Lakeshore Road, Ste-Anne-de-, Bellevue, QC H9X 3V9 Canada; 5 McGill School of Environment, 3534 University Street, Montreal, QC H3A 2A7 Canada

## Abstract

The Northern Biodiversity Paradox predicts that, despite its globally negative effects on biodiversity, climate change will increase biodiversity in northern regions where many species are limited by low temperatures. We assessed the potential impacts of climate change on the biodiversity of a northern network of 1,749 protected areas spread over >600,000 km^2^ in Quebec, Canada. Using ecological niche modeling, we calculated potential changes in the probability of occurrence of 529 species to evaluate the potential impacts of climate change on (1) species gain, loss, turnover, and richness in protected areas, (2) representativity of protected areas, and (3) extent of species ranges located in protected areas. We predict a major species turnover over time, with 49% of total protected land area potentially experiencing a species turnover >80%. We also predict increases in regional species richness, representativity of protected areas, and species protection provided by protected areas. Although we did not model the likelihood of species colonising habitats that become suitable as a result of climate change, northern protected areas should ultimately become important refuges for species tracking climate northward. This is the first study to examine in such details the potential effects of climate change on a northern protected area network.

## Introduction

Protected area networks are our most valuable resource for *in situ* conservation of global biodiversity^1^, therefore many studies have tried to project future impacts of climate change on protected areas^2,3^. Indeed, whereas protected areas are remarkably successful at buffering species from habitat loss and fragmentation, they will not protect them from climate change^4^. The most obvious cases are those of endemic or threatened species for which climate conditions would become unsuitable in protected areas where they currently occur^2,5^. In other cases, climate change could substantially reduce suitable climate space of species inside protected area networks, potentially driving them to become endangered^6^. Other impacts of climate change on protected areas include the potential colonization of invasive or pest species^7,8^, since protected areas may facilitate their range expansion^9^. Significant disruption of current species assemblages has also been predicted inside protected areas^3,6^. These impacts of climate change challenge our definition of the protected area’s concept. The efficacy of protected area networks in preserving future biodiversity is still an unresolved issue, as various indicators suggest that protected areas may become less effective in the future^10,11^, while others suggest that rigorously defined networks can play a key role in mitigating the worst impacts of climate change on biodiversity^3,12^.

Increased rates of species turnover have often been projected as a result of climate change^13,14^, and many species may face a considerable reduction of their suitable climate space, potentially facing extinction^15,16^. Paradoxically however, many populations from northern latitudes could benefit from global warming if they are located at the northern range boundary of the species and limited by cold climate conditions. For example, in temperate regions of the northern hemisphere, ectothermic species like amphibians might expand their distribution in response to warming if dispersal of individuals is allowed^17^. This expected northward expansion of species ranges in the northern hemisphere and the potential increase of regional species richness that this might incur have been coined as the Northern Biodiversity Paradox^18^. In support of this hypothesis, several large scale studies have highlighted a potential increase in species richness in northern and temperate regions^2,13,19^, and a few have specifically focused on regions where biodiversity could potentially increase due to climate change^20–22^.

In this paper, we assessed the potential impacts of climate change on the biodiversity of a network of 1,749 protected areas spread over >600,000 km^2^ in Quebec, Canada. Our study area is characterized by a northern climate with cold winters and short summers, encompasses the northern range boundary of many species, and might be exposed to high rates of climate displacement^23^. We predicted that many species would see their suitable climate space expanding inside the study area as climate warms during this century, leading to potential species gain and turnover within protected areas. We used changes in the potential probability of occurrence of 529 species of birds, amphibians, trees, and other vascular plants, for which the ecological niche was modeled, to evaluate potential impacts of climate change on (1) species gain, loss, turnover, and richness in protected areas, (2) extent to which regional species diversity is represented in protected areas (representativity of protected areas), and (3) extent of species ranges located in protected areas. We discuss the implications of our findings for biodiversity conservation and protected area management in cold climate regions.

## Results

### Potential impacts of climate change in protected areas

#### Species gain, loss, turnover and richness

**Figure 1:**
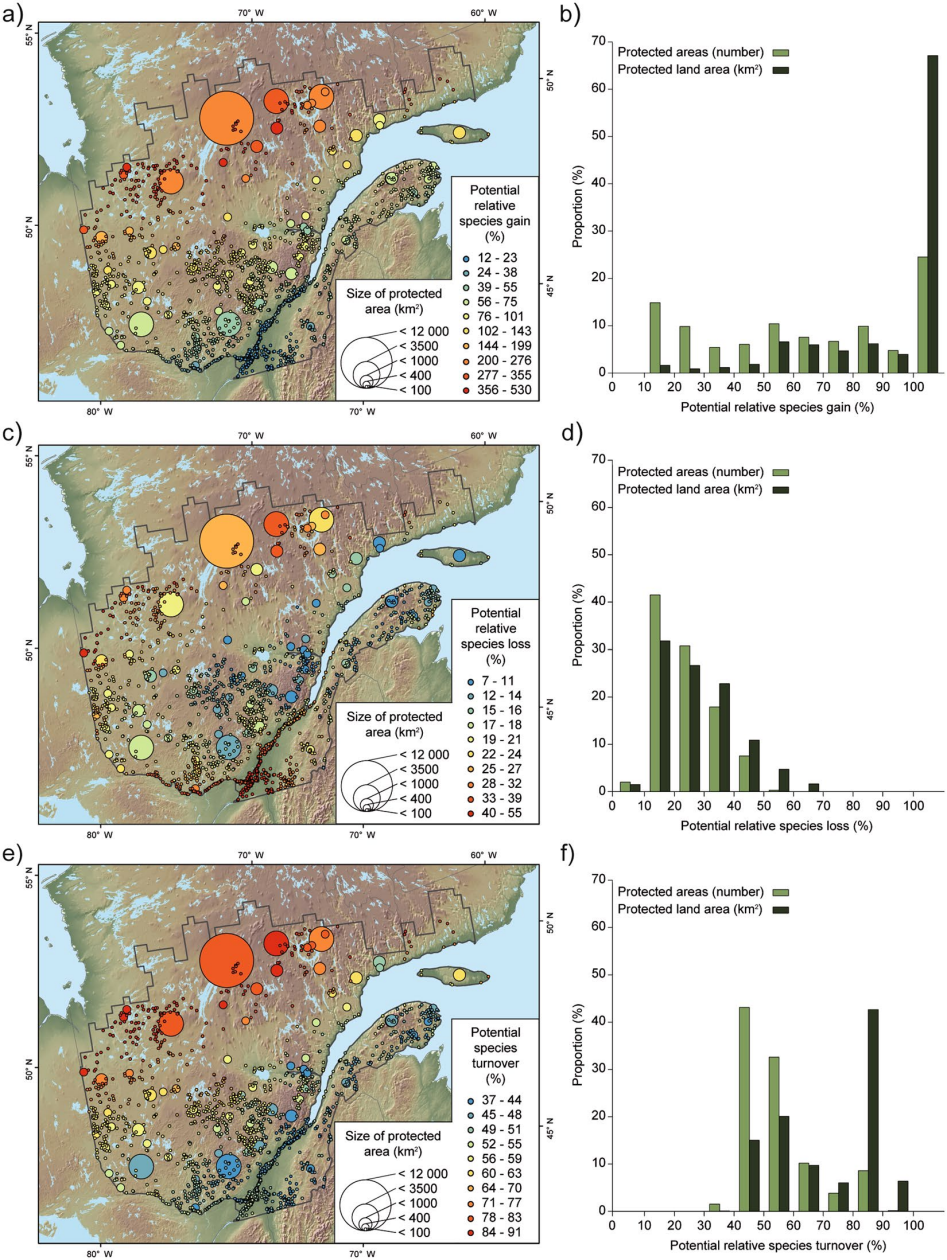
Potential climate change impacts on biodiversity of protected areas of southern Quebec, Canada, for 2071–2100. Biodiversity was assessed through analysis of 529 species of birds, amphibians, trees, and other vascular plants. Potential impacts are illustrated through potential relative species gain (*rG*, top panels), potential relative species loss (*rL*, middle panels), and potential species turnover (*T*, bottom panels). Relative species gain and loss are expressed as a percentage of the modeled species richness for the 1961–1990 reference period. Maps show spatial patterns of *rG* (panel a), *rL* (panel c), and *T* (panel e). Bar charts show in pale green the proportion of protected areas (n = 1,749) and in dark green the proportion of total protected land area (n = 42 676 km^2^) for each value class of *rG* (panel b), *rL* (panel d), and *T* (panel f). Note that in panel b, bars located on the right end of the X axis gather potential relative species gains ≥100%. Maps were created using ESRI ArcGIS 9.4 (http://www.esri.com/).

In 2071–2100, climate is expected to become suitable for many new species in protected areas of Quebec. We project a potential relative species gain ranging from 12 to 530% (92% ± 93), depending on the protected area (Fig. 1b, pale bars). This effect is maximized in northernmost protected areas (Fig. 1a). Because many large protected areas are located in northern parts of the study area, 67% of the total protected land area could experience a relative species gain ≥100% (Fig. 1b, dark bars) if species distributions were to track predicted changes in climatic conditions.

However, many species could also lose suitable climate space in protected areas, especially in the southernmost protected areas and, to a lesser extent, in northern and western areas (Fig. 1c). The potential relative species loss ranges from 7 to 55% (24% ± 10) depending on protected areas (Fig. 1d, pale bars), and is >50% on 6% of the total protected land area (Fig. 1d, dark bars).

Locally, model results suggest that climate change may impose a strong pressure on species composition, with a potential species turnover ranging from 37 to 91% (55% ± 12) depending on protected areas (Fig. 1f, pale bars). This effect is clearly stronger at higher latitudes (Fig. 1e). Overall, 49% of total protected land area might experience a species turnover ≥80% (Fig. 1f, dark bars), according to the results generated from the 529 studied species.

**Figure 2:**
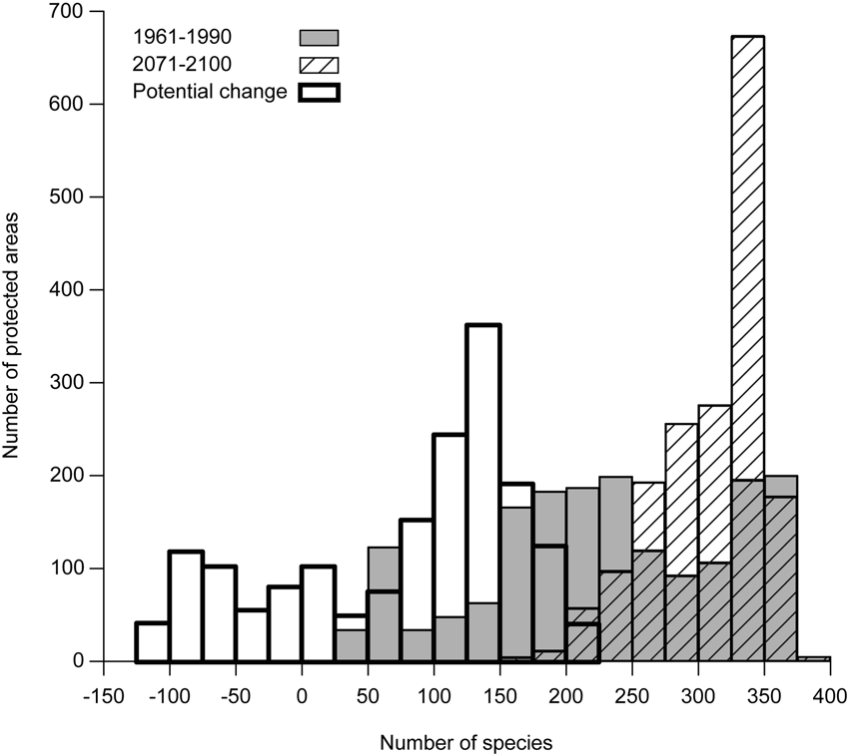
Frequency distribution of modeled species richness in 1,749 protected areas of southern Quebec, Canada. A total of 549 species were modeled. The periods 1961–1990 and 2071–2100 are represented as grey and hatched bars, respectively. The frequency distribution of the potential change in modeled species richness between 1961–1990 and 2071–2100 is represented by bars with thick borders.

Protected areas would contain in general more species in 2071–2100 than in 1961–1990 (V = 161 903, p-value < 0.001, n = 1749) if all species tracked their suitable climatic conditions. This is shown in Fig. 2 by the shift to the right of the frequency distribution of species richness between 1961–1990 (blue bars) and 2071–2100 (red bars). Despite this general tendency, however, species richness might decrease in 401 protected areas (23%), as shown by the thick-border bars with negative changes in number of species (Fig. 2).

#### Representativity of protected areas

**Figure 3:**
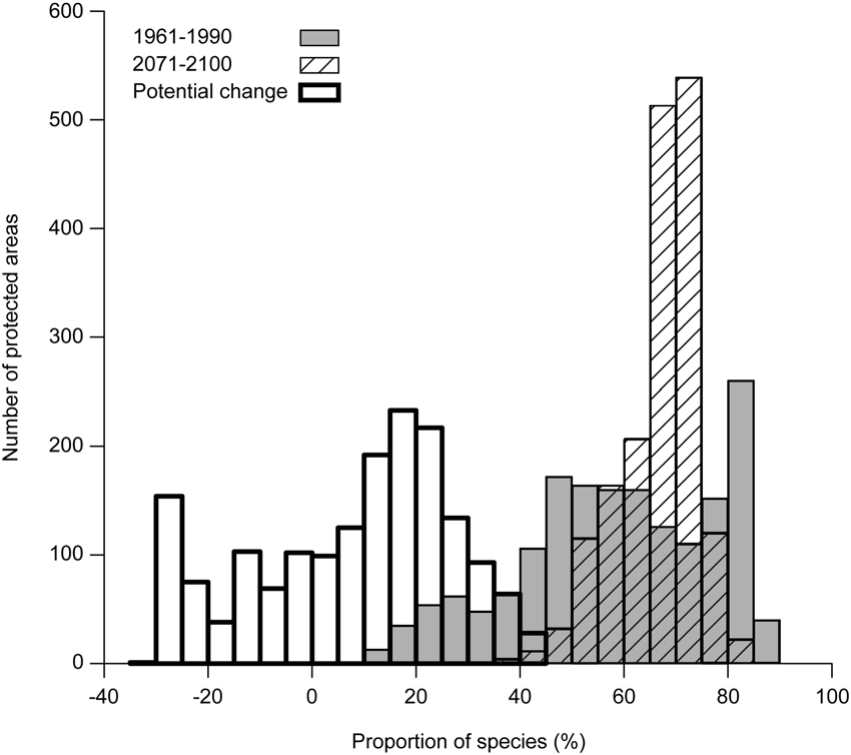
Frequency distribution of potential representativity of 1,727 protected areas of southern Quebec, Canada. The periods 1961–1990 and 2071–2100 are represented as grey and hatched bars, respectively. Representativity is defined as the proportion of species that occur in the cells containing a given natural province (as defined in Quebec’s Ecological Reference Framework) that also occur in the cells containing a protected area located in that natural province. The frequency distribution of the potential change in potential representativity between 1961–1990 and 2071–2100 is represented by bars with thick borders.

Representativity of protected areas assessed at the scale of natural provinces would also generally increase in 2071–2100 compared to 1961–1990 (V = 424 220, p-value < 0.001, n = 1727) if all species were to track their suitable climatic conditions. This is shown in Fig. 3, where the frequency distribution of representativity slightly shifts to the right between 1961–1990 (blue bars) and 2071–2100 (red bars). The representativity of a majority of protected areas would thus increase, although it would decrease in 542 (31%) of them (thick-border bars in Fig. 3). Results are very similar when analyses are done at the coarser scale of the study area or at the finer scale of natural regions (Supplementary Figs S1 and S2).

### Extent of species range located in protected areas

**Figure 4:**
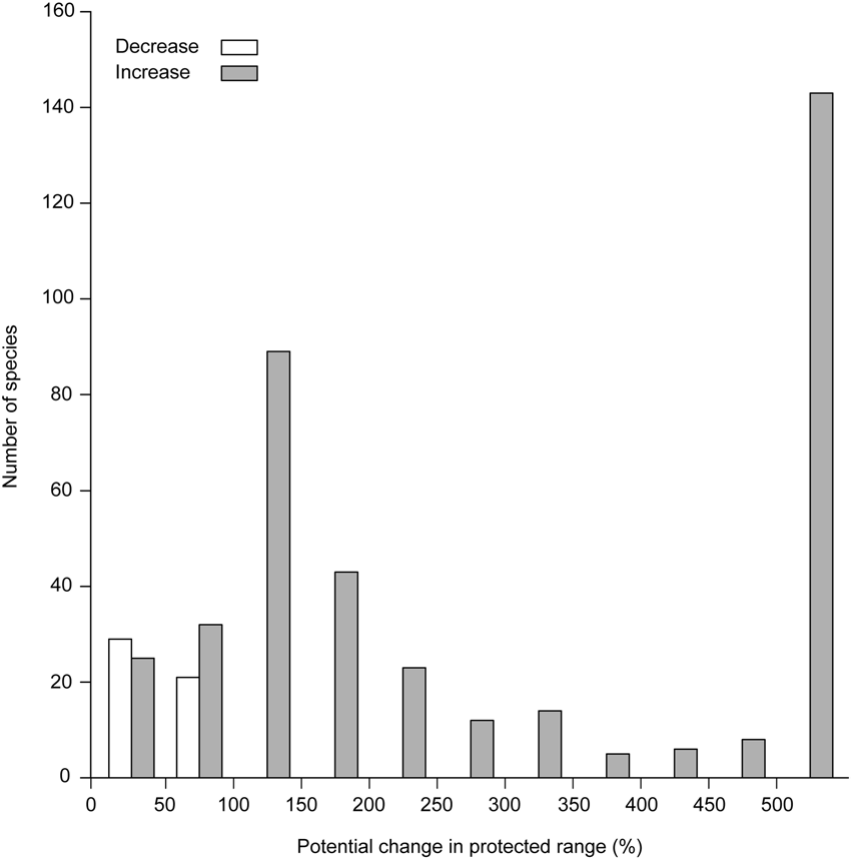
Number of species in each class of potential change in extent of species range located in protected areas. N = 450 species. Potential change in extent of species protected range is defined as the change in the protected land area overlapping with the modeled species distribution that is projected to occur between 1961–1990 and 2071–2100, expressed as a percentage of the protected surface a species is projected to occur in during 1961–1990. Grey bars show increases and white bars show decreases. Of the 529 species studied, 79 were excluded from the analysis because their modeled distribution in 1961–1990 did not overlap the study area.

The level of protection provided by protected areas could substantially increase for most of the studied species, again if they are to track predicted changes in climatic conditions. The potential changes in extent of species protected range vary from −100% to 1.7 × 10^6%^ (median = 177% ± 239 MAD; Fig. 4) according to our simulations. Fifty out of 450 species might however experience a decrease in the extent of their protected range. Supplementary Table S1 gives the species-specific details regarding the potential change in level of protection provided by the protected areas.

## Discussion

Climate conditions in Quebec will become more suitable to many species from eastern North America. These species could therefore expand their ranges northward, in agreement with modeling results or observations previously conducted in northern and temperate regions for various plants^22,24^, birds^25,26^, mammals^20,21^, and arthropods^27^. In a context of decreasing species richness with latitude^28^, northern protected areas could experience important species gains, as expected from the Northern Biodiversity Paradox. The extent of species ranges located in protected areas should generally increase, despite heterogeneity among species. Similarly, despite heterogeneity among protected areas, representativity of protected areas should generally increase, whether this is assessed at the coarse scale of the study area, at the intermediate scale of natural provinces or at the fine scale of natural regions.

Some of our methods may have underestimated the changes to come. For instance, among plants other than trees, only species native of Quebec were modeled, so no immigration from the U.S.A. was allowed for this group and the potential northward influx of species is actually higher than presented. Before investigating the conservation implications of these predicted range expansions into northern protected areas, we highlight the important topic of dispersal limitations, we raise the questions of local extirpations and rupture of present biotic interactions, and we recognize a caveat regarding the interpretation of results from small protected areas.

We are aware that species will not necessarily fully track their new climatic conditions, as several factors can impede or delay immigration of species in protected areas of Quebec. First, dispersal rates could be 1.5 to 3 km/year for birds and 0.1 to 0.5 km/year for amphibians and plants, respectively^16^. However, the velocity of shift for the 5 °C isotherm is projected to be about 2 km/year in Quebec during this century^18^. Insufficient dispersal capacities of species will thus delay immigration in response to climate change. In addition, natural and anthropogenic habitat fragmentation can further impede dispersal rates of organisms by reducing connectivity between suitable patches^29,30^. For example, the St. Lawrence River which divides southern Quebec from east to west, the urbanized area of Montreal, and the fragmented agro-forested habitats of Southern Quebec all represent major barriers to species dispersal. Substantial lags between climate change and resulting immigration were shown for butterflies in Great Britain^31^, forest-understory plants in U.S.A.^32^, trees in western North America^33^, and birds in North America^34^ and France^35^. Second, competition with presently-established species can strongly limit colonization by newcomers^36^. For example, persistence of long-lived conifers in the boreal biome could delay the northward expansion of deciduous trees, which could in turn delay the expansion of birds associated with mature deciduous stands. More generally, we ignored many variables other than climate in defining future habitat suitability of species, therefore our predictions for future suitable habitat are sometimes overly optimistic^37^. Colonization requires sequential successes in the dispersal, establishment, and survival of individuals on new suitable sites as well as growth and persistence of populations via continued reproduction^38^. Immigration of species is delayed if any of these steps is hampered. As a consequence, our results are not meant to offer quantitative assessment of local changes in biodiversity within a specific time frame, but only to provide the best-available indication of the strong pressure that climate change will impose on biodiversity.

Potential species gains, however impressive they are, should not draw attention away from potential local extirpations of species that may no longer find suitable conditions in protected areas where they currently occur. The geographical pattern of potential relative species loss suggests that several species could face local extirpations in the southernmost protected areas of Quebec. However, relative species loss could also be important in protected areas located at higher latitudes, where only few local extirpations can have drastic effects on simple communities^39^. Moreover, sampling bias in the modeling of birds (species breeding north of 53°N were excluded from ecological niche modeling) may mask more important species losses in the northernmost areas. Predicted reduced breeding range by climate change has been demonstrated for bird species adapted to cold climates in subarctic and arctic Europe^40^. These potential extirpations are of great concern for conservation because protected areas are often specifically designed to preserve vulnerable species from human threats, and we demonstrate that such species can be pushed out of protected areas by climate change.

As shown by our results, climate change could lead to major changes in the composition of current biological communities in protected areas of Quebec. This agrees with recent findings suggesting important potential species turnover for North American boreal forest birds^41^. Greater turnover rates are expected in the northernmost protected areas, where relatively simple communities could be highly affected by a proportionally high number of immigrant species. Changes in community structure and composition may affect species-mediated ecological processes^42^, lead to a rupture of present biotic interactions^43^, or have indirect effects on other species via trophic cascades^44,45^. Species that do not currently coexist may do so in the future^46^. Predicting the outcomes of new interactions in these novel communities is a major scientific challenge.

We recognize that we overestimated the richness and representativity of small protected areas because we assigned to these protected areas the values of the 20 km × 20 km cell in which they were contained. This may not impact general comparisons across time horizons, but one should resist the temptation to interpret results at the scale of single protected areas.

### Conservation implications

The protected areas of Quebec are poised to becoming biodiversity refuges of continental importance, which has four imbricated conservation implications. First, the efficiency of the Quebec network of protected areas in preserving biodiversity could be compromised by limitations to species dispersal. A biodiversity deficit could occur in some areas of Quebec if many species are trapped for decades or centuries between rapid retreat at their southern edge and slow advance at their northern edge^38^. Therefore, increasing connectivity between protected areas and preserving and restoring potential immigration corridors are priorities.

Second, colonizing species favour protected over unprotected sites^9^ and managers of protected areas in northern regions will have to deal with an increasing number of new immigrant species^47^. Newly arriving species can impact negatively ecosystem structure and function^48^. At the same time, self-sustaining populations of non-native species could become necessary in some protected areas to ensure local ecosystem functions and services if historical communities are deeply modified^49^. In this context, deciding which new species should be controlled and which should be tolerated or favored will represent an immense challenge.

Third, in Canada as in several other high-latitude countries, northern peripheral species are already a significant portion of species at risk^50^. These species can have negative impacts on native communities locally, but from a wider point of view, genetic diversity of leading-edge peripheral populations may help species to cope with climate change^50^. Hence, assigning conservation status to rare and recently naturalized species is a thorny issue, and conservation value of rare new species should be considered in a long-term continental perspective rather than short-term national perspective.

Fourth, the important species turnover expected in northern protected areas emphasizes the hopelessness of trying to preserve a snapshot of today’s biodiversity. This challenges the traditional paradigm of conserving the ecological integrity of National Parks. Designing conservation to preserve site resilience and a diversity of physical features and abiotic conditions that are associated with ecological diversity could be a valuable biodiversity conservation strategy under climate change^51,52^.

Our study is the first to examine in such details the potential effects of climate change on the biodiversity of a large network of northern protected areas. As predicted by the Northern Biodiversity Paradox, implications of our results entail some shifts in conservation paradigms. These paradigm shifts, although largely familiar to climate change biologists, have yet to be translated at the local scale by practitioners into short and long-term action plans.

## Methods

### Study design

We used ecological niche modeling to forecast the potential effects of 21^st^ century climate change on the distribution of a large set of species. These species currently occur in the studied network of protected areas or could potentially be found there in the future as climate warms up. We used 1961–1990 as our reference period and 2071–2100 as future time horizon. The project builds on a large collaborative effort (the CC-Bio Project) to assess the effects of climate change on Quebec biodiversity in general^18,53^, and on Quebec forests in particular^24,37,54^.

### Study area

We studied the protected area network of the southern part of the province of Quebec, Canada. The study area extents from 45°N to 53°N (Supplementary Fig. S3) and is located inside the temperate broadleaf/mixed forest and boreal forest/taiga biomes^55^. We studied 1749 protected areas, or 72% of the 2439 protected areas found in Quebec. Quebec protected areas located totally or partially outside the study area (n = 148) and marine/coastal protected areas (n = 542) were excluded from the analyses. The studied protected areas represent 32% (43,689 km^2^) of the surface covered by the Quebec protected area network, and 95% of the protected areas studied are less than 50 km^2^ in size (Supplementary Fig. S4). Our analyses cover a wide range of IUCN protected area categories (Supplementary Table S3), with categories IV (37%) and VI (30%) being the most represented (Supplementary Fig. S4).

### Species and environmental data

We collected presence/absence or occurrence data (1961–1990) on 176 species of birds, 40 species of amphibians, 90 species of trees, and 223 other vascular plants (total 529 species) in a large area (hereafter, the modeling area) covering most of eastern North America (see Supplementary Table S1 for the complete list of species and Supplementary Fig. S3 for a map of the study area). We then reported this information on the 9,806 20 km x 20 km grid cells of the modeling area.

Species data come from several Canadian and U.S. governmental survey databases and from large amateur observation programs, as detailed in Supplementary Methods: Species and environmental data.

We excluded bird species breeding north of 53°N because data were missing from a large fraction of their breeding range. We lacked distribution data for amphibians and vascular plants from Ontario, located south-east of Quebec, where some future immigrants to Quebec potentially occur. For vascular plants other than trees, we modeled only species already present in the province of Quebec. These sampling constraints generated some biases that we have raised in Discussion.

Although we studied only a fraction of the regional biodiversity, the selected species offer a good representation of the different types of species ranges (e.g., northern peripheral, subcosmopolitan), they have a diversified natural history (e.g., plants, herbivores, predators), they include species that strongly structure ecosystems (e.g., trees), and some of them are of conservation or management importance. Our results should thus be highly informative regarding the potential spatial reorganization of regional biodiversity.

Three to four climate variables were used to relate species distribution to climate, depending on taxon (Supplementary Table S5). These variables were selected for their biological relevance and according to a correlation matrix selection, such that the Pearson correlation coefficient was under 0.6 for all pairs of selected variables. The origin of climate data for the reference and future periods is detailed in Supplementary Methods: Species and environmental data.

Soil and topographic conditions influence distribution patterns for many plant species^37^. Surface deposits (7 classes) and soil drainage conditions (3 classes) were used to model trees following Chambers *et al*.^54^. Sources for soil and topographic data are given in Supplementary Methods: Species and environmental data.

### Ecological niche modeling

Ecological niche models were performed for each species using up to eight statistical approaches within an ensemble forecasting framework^56,57^ in order to consider uncertainty due to differences in modeling and projection procedures. Analyses were performed using the BIOMOD package^58^ implemented in the R statistical software^59^. Details on BIOMOD implementation are available in Supplementary Methods: Ecological modeling, which also describes how the predictive performance of models was evaluated and the multiple projections summarized.

Pseudo-absences were generated to train ecological niche models, except for trees for which real absences were available. For each species, a spatial buffer was overlaid around all cells reporting species presence, and randomly selected absences were chosen outside this buffer^25^. To give equal weight to presences and pseudo-absences, we selected the same number of pseudo-absences as we had presences for a given species (prevalence = 0.5). The pseudo-absence selections were repeated ten times. We provide the distribution of AUC values for calibrated models in Supplementary Fig. S5.

### Analyses: Species gain, loss, turnover and richness in protected area

Species absent in 1961–1990 from a cell where climate would become suitable in 2071–2100 could potentially colonize that cell, thus resulting in a distribution gain. Conversely, species present in 1961–1990 in a cell where climate would become unsuitable may become locally extirpated, thus incurring a distribution loss. We calculated for each cell the number of species for which climate should become suitable (potential gain, *G*) or unsuitable (potential loss, *L*) in 2071–2100, and divided these values by species richness calculated from modeled distributions during the reference period (*ref SR*) to obtain a potential relative species gain (*rG*) or loss (*rL*), following equations (1) and (2). We also calculated for each cell the potential species turnover (*T*) using equation (3) from Peterson *et al*.^60^. *T* ranges from 0 (species composition does not change between 1961–1990 and 2071–2100) to 100 (full species turnover).

1 $$rG=100\times G/ref\,SR$$

2 $$rL=100\times L/ref\,SR$$

3 $$T=100\times (G+L)/(ref\,SR+G)$$

When a protected area was contained in a single cell, the gain, loss, turnover, and richness values of that cell were assigned to the protected area. When a protected area overlapped several cells, these cells were aggregated into the smallest polygon containing the protected area, and values were calculated for this polygon before being assigned to the protected area. This allowed us to report results with protected areas as sampling units.

Protected areas differ in their conservation importance due to their varying size and degree of overlap with other protected areas. We thus also report results with reference to protected land area. To do this we calculated for each cell how much land was protected, and assigned to this land the values of the cell. This allowed us to assess how much protected land area within the studied network of 1749 protected areas could be attributed to various classes of species gain, loss, turnover, and richness.

### Analyses: Representativity of protected areas

We estimated the representativity of each protected area by dividing its species richness by the species richness of the region to which the protected area belongs, using modeled distributions to assess species richness (see above). Representativity equals 1 when all species predicted to occur in the region are also predicted to occur in the cells containing the protected area. We repeated the analysis for 1961–1990 and 2071–2100 as well as at three spatial scales, i.e. the whole study area, natural provinces, and natural regions. Natural provinces and natural regions are levels I and II of Quebec’s Ecological Reference Framework^61^ and are used as reference to plan Quebec’s network of protected areas. The scale of natural provinces matches roughly that of the level III ecoregions of North America.

### Analyses: Extent of species range located in protected areas

We evaluated how climate change could potentially affect the level of protection offered by protected areas to each modeled species. We excluded from the analysis 79 species (19 birds, 18 amphibians, 41 trees, and 1 other vascular plant) for which the modeled distribution in 1961–1990 did not overlap the study area. For each species, we defined the potential change in protected range (Δ*PR*) between 1961–1990 (*ref PR*) and 2071–2100 (*future PR*) as the change in protected land area overlapping the modeled species range, assuming species fully track their suitable climatic conditions. We calculated Δ*PR* using equation (4). Δ*PR* is positive when the extent of the species range located on protected land increases and negative when it decreases.

4 $${\rm{\Delta }}PR=100\times (future\,PR-ref\,PR)/ref\,PR$$

### Statistics and reporting

We expressed measures of central tendency as mean ± SD, except when extreme values make the use of median ± MAD (median absolute deviation) more appropriate. We used Wilcoxon signed-rank tests (data were not normally distributed) to compare richness and representativity of protected areas across time horizons. We did analyses and statistical tests using R version 2.14.1^59^ and maps using ArcMap version 10.0^62^.

### Data availability

Web links for publicly available datasets are given in Supplementary Table S4. Detailed modeling results (3,405 tables) and observed and modeled distributions (8,172 maps accessible via drop-down menus) for the studied species (except trees) are available at http://cc-bio.uqar.ca/english/en_atlas.html. Occurrence data and modeled distributions for trees are available at http://mffp.gouv.qc.ca/changements-climatiques/outil/telecharger.html.

## Electronic supplementary material

Supplementary information
