# Supplementary material for: Northern protected areas will become important refuges for biodiversity tracking suitable climates
Source: Sci Rep. 2018 Mar 15;8:4623. doi: 10.1038/s41598-018-23050-w (PMC5854666; doi:10.1038/s41598-018-23050-w)
Supplement: Supplementary file 1 — Supplementary information [file 41598_2018_23050_MOESM1_ESM.pdf]

Supplementary information for

Northern protected areas will become important refuges for biodiversity tracking suitable climates

Dominique Berteaux, Marylène Ricard, Martin-Hugues St-Laurent, Nicolas Casajus, Catherine Périé, Frieda Beauregard & Sylvie de Blois

Table of contents

Supplementary Methods: Ecological niche modeling 3

Supplementary Methods: Species and environmental data 5

Supplementary Table S1. List of the 529 species included in the analysis of climate change impacts on the biodiversity of protected areas in southern Quebec, Canada, between 1961-1990 and 2071-2100, and species-specific data are given regarding the potential change in level of protection provided by the protected areas............................ 8

Supplementary Table S2. Number of repetitions for various steps of ecological niche modeling. 32

Supplementary Table S3. Definitions and purposes of IUCN protected area categories. 33

Supplementary Table S4. Web links of publicly available sources of data on species, climate, soil, and topography used to assess the impacts of climate change on the biodiversity of a northern network of protected areas in southern Quebec, Canada. 34

Supplementary Table S5. Climatic variables used to calibrate ecological niche models for different groups of species. 35

Supplementary Figure S1. Frequency distribution of potential representativity of 1,749 protected areas of southern Quebec, Canada (analysis at the scale of the study area). 36

Supplementary Figure S2. Frequency distribution of potential representativity of 1,711 protected areas of southern Quebec, Canada (analysis at the scale of the natural region). 37

Supplementary Figure S3. Protected areas included in and excluded from the study area located in southern Quebec, Canada. 38

Supplementary Figure S4: Proportion of protected areas in each IUCN category according to size of protected areas in the study area. 39

Supplementary Figure S5: Distribution of AUC values for the 158,920 models calibrated in this study. 40

References 41

Supplementary Methods: Ecological niche modeling

We used the BIOMOD package[1] implemented in the R statistical software[2] to perform ecological niche modeling using the following statistical approaches (from 6 to 8 depending on taxon) and settings:

- Generalized linear models
  - Quadratic form of predictors
  - Internal stepwise selection of predictors based on AIC
  - Logit link function
- Generalized additive models
  - GAM performed using the package gam
  - Maximum smooth term = 4
  - Logit link function
- Classification tree analyses
  - Default parameters
- Random forests
  - Classification approach
  - 500 trees per model
- Generalized boosting models
  - Bernouilli distribution of error
  - 2500 trees per model
  - 3 cross-validations with a 0.5 bag fraction
- Multiple adaptive regression splines
  - Default parameters
- Artificial neural networks (used for trees only)

- 5 cross-validations based on AUC
- 200 iterations to optimize parameters
- Weight decays of parameters optimized by cross-validation

- Flexible discriminant analyses (used for birds, amphibians and trees)
  - Based on the MARS method

We evaluated the predictive performance of models using the area under the curve (AUC) of the receiver-operating characteristic (ROC) plot[3], and used a split-sample procedure to assess predictive performances on pseudo-independent data[4]. This split-sample procedure randomly split presence/absence data in two datasets, where 70% of the initial data were used to calibrate models whereas the remaining 30% were used to evaluate predictive performances. The evaluation step was repeated 10 to 20 times depending on the taxon (Supplementary Table S2 online).

Due to spatial autocorrelation[5,6] and underlying spatial structure of data[7], random cross-validation produces overly-optimistic assessments of model error, particularly when projecting to new climates. A recent solution to this bias is to cross-validate models using a spatially independent approach, such as buffered leave-one-out or k-fold with blocking[6,7]. Unfortunately our modeling was performed before these solutions were published.

From 80 to 140 models were projected in 2071-2100 under 4 to 7 selected climate change scenarios, depending on the taxonomic group (Supplementary Table S2 online). This resulted in 480 to 980 projections into the future (Supplementary Table S2 online), that we summarized using a consensus technique[8] which allowed us to summarize information while considering uncertainty. We aggregated probabilities of occurrence

(the raw output of the models) for the reference period and for 2071-2100 using the weighted average approach and AUC as the weight[9]. Finally, we converted these probabilities into presence/absence using the sensitivity-specificity sum maximization approach[10]. This approach uses a threshold that maximizes the good classification of both presences and absences. We are aware that this approach can be biased when prevalence is unbalanced, but we selected the same number of pseudo-absences as the number of presences. Therefore for each species (except for trees) the prevalence in original datasets was 0.5, and during the split-sample step, we forced the calibration datasets to have the same prevalence as the original dataset. We used the same threshold for the reference and future time horizons, so that any bias, if present, would be constant.

Supplementary Methods: Species and environmental data

Species data **–** Species data come from the following sources. **Birds**: North American Breeding Bird Survey, a standardized breeding season roadside survey; *Étude des Populations d'Oiseaux du Québec*[11], a compilation program of observations from amateur birders in Quebec. **Amphibians**: *Atlas des Amphibiens et Reptiles du Québec*, a checklist compilation program that gathers observations from amateur herpetologists in Quebec; National Amphibian Atlas, which provides species distribution data for the U.S. at the county level; Atlantic Canada Conservation Data Center, which provides information about species distribution for New Brunswick, Canada. **Trees**: the 3rd decennial forest inventory of the *Ministère des Forêts, de la Faune et des Parcs* (MFFP) of Quebec, an extensive dataset of >95,000 forest plots reporting the presence/absence of all tree species; USDA Forest Service Tree Atlas website, a compilation from >100,000 forest inventory and analysis plots assessed by the USDA Forest Service[12] and giving the

occurrence of trees in the U.S. portion of our modeling area. **Other vascular plants**: all major Eastern North-American herbaria with digitized records; the USDA plants database; the Environment Canada Biodiversity Portrait of the St Lawrence, which encompasses a detailed floral survey along the shores of the St Lawrence River; the *Point d'Observation Écologique* of the MRNF, an inventory with >28,000 plots. The coverage of each species was compared against the Flora of North America[13] and Rousseau[14] for their completeness.

Climate data – Climate for 1961-1990 was obtained from the U.S. Forest Service Rocky Mountain Research station website[15] by deriving raw climatic surfaces for total precipitation and for maximum, minimum, and average temperatures. Climate change scenarios for 2071-2100 originated from 15 global climate models made available by the World Climate Research Programme's Coupled Model Intercomparison Project phase 3[16]. These climate models were run one or several times under three projected greenhouse gas emissions scenarios (SRES family A2, A1B and B1[17]). Climate change scenarios obtained from the high-resolution Canadian regional climate model run under the SRES family A2 were also used[18]. This made available to us 70 climate change scenarios which were downscaled to the 20 km x 20 km modeling grid using linear triangle-based interpolation[19]. Since our analyses were done, IPCC's AR5[20] has selected new climate models and new forcing scenarios. However, the global mean temperature response simulated by AR5 models is very similar to that simulated by the preceding models[21]. Projecting a large number of species under 70 climate change scenarios is computationally intensive. We thus selected a representative subset using a multivariate clustering approach[22]. This method retains the coverage of uncertainty in future climatic

conditions while minimizing the number of climate change scenarios used. As this method depends on the variables used in species distribution models, the number of climate change scenarios selected varies with taxa, from four to seven.

Soil and topographic data – Edaphic data were obtained from the Soil Survey Geographic (SSURGO) Database (version 2.1) for the U.S. portion and the MFFP for the Canadian portion. Elevation data come from the USDA Forest Service tree Atlas used by Iverson et al.[23] for the U.S.A., and from the Canadian Digital Elevation Data.

Data availability is indicated at the end of the Methods section.

Supplementary Table S1. List of the 529 species included in the analysis of climate change impacts on the biodiversity of protected areas in southern Quebec, Canada, between 1961-1990 and 2071-2100, and species-specific data are given regarding the potential change in level of protection provided by the protected areas. Number of protected areas and protected range referred as protected areas or protected land area overlapping with the 20 x 20 km cells belonging to the species distribution. Potential future distributions assume that species track their climate envelope with no time lag. Anticipated losses appear in bold. SD = Standard deviation. MAD = Median absolute deviation.

Species	Number of protected areas located in the species distribution		Potential change in number of protected areas	Protected range (km^2)		Potential change in protected range
	Modelled distribution 1961-1990	Modeled distribution 2071-2100		Modelled distribution 1961-1990	Modelled distribution 2071-2100	
Birds (n = 176)						
Accipiter cooperii	522	1581	1059	2343	20 368	769
Accipiter striatus	1336	1690	354	15 292	39 897	161
Aegolius acadicus	477	654	177	4109	25 392	518
Agelaius phoeniceus	1510	1749	239	18 247	38 248	110
Aix sponsa	1195	1744	549	12 008	35 473	195
Ammodramus henslowii	83	919	836	226	9 001	3878
Ammodramus savannarum	357	1522	1165	1173	15 927	1257
Anas americana	1070	573	**-497**	8817	25 893	194
Anas clypeata	666	195	**-471**	4164	11 600	179
Anas discors	1359	1735	376	13 653	36 059	164
Anas platyrhynchos	1377	1740	363	14 006	34 440	146
Anas strepera	721	232	**-489**	4281	10 327	141
Archilochus colubris	1353	1747	394	14 311	36 551	155
Ardea alba	52	418	366	160	1019	537
Ardea herodias	1494	1749	255	16 796	38 441	129

Species	Number of protected areas located in the species distribution		Potential change in number of protected areas	Protected range (km^2)		Potential change in protected range
	Modelled distribution 1961-1990	Modeled distribution 2071-2100		Modelled distribution 1961-1990	Modelled distribution 2071-2100	
Aythya affinis	803	318	**-485**	5385	18 936	252
Baeolophus bicolor	0	1048	1048	0	10 443	-
Bartramia longicauda	835	1717	882	4629	31 374	578
Bombycilla cedrorum	1534	1749	215	19 293	42 339	119
Bonasa umbellus	1530	1747	217	19 161	42 579	122
Botaurus lentiginosus	1562	1749	187	19 376	42 583	120
Bucephala albeola	1168	433	**-735**	11 696	24 509	110
Buteo jamaicensis	1380	1747	367	15 442	38 077	147
Buteo lineatus	713	1689	976	3822	27 338	615
Buteo platypterus	1513	1648	135	17 586	40 733	132
Butorides virescens	659	1701	1042	2813	26 802	853
Caprimulgus vociferus	739	1696	957	3822	25 855	576
Cardinalis cardinalis	270	1497	1227	634	17 025	2587
Carduelis pinus	1576	1274	**-302**	20 447	41 015	101
Carduelis tristis	1497	1749	252	17 797	37 554	111
Carpodacus mexicanus	493	1631	1138	1395	22 906	1542
Carpodacus purpureus	1590	1689	99	20 649	42 375	105
Cathartes aura	590	1602	1012	2095	19 556	833
Catharus fuscescens	1503	1749	246	17 605	42 339	140
Catharus guttatus	1614	1551	**-63**	23 018	42 066	83
Catharus ustulatus	1617	1286	**-331**	23 327	41 148	76
Certhia americana	1444	1644	200	16 380	41 773	155
Chaetura pelagica	1281	1743	462	13 195	35 572	170

Species	Number of protected areas located in the species distribution		Potential change in number of protected areas	Protected range (km^2)		Potential change in protected range
	Modelled distribution 1961-1990	Modeled distribution 2071-2100		Modelled distribution 1961-1990	Modelled distribution 2071-2100	
Charadrius vociferus	1481	1749	268	17 640	37 554	113
Chlidonias niger	701	625	**-76**	2553	17 191	573
Chondestes grammacus	0	0	0	0	0	-
Chordeiles minor	1404	1736	332	15 214	35 746	135
Circus cyaneus	1460	1746	286	15 661	39 245	151
Cistothorus palustris	574	1557	983	2033	21 155	941
Cistothorus platensis	596	1417	821	2476	24 577	893
Coccothraustes vespertinus	1579	1133	**-446**	20 233	39 721	96
Coccyzus americanus	516	1657	1141	1791	22 351	1148
Coccyzus erythropthalmus	1159	1737	578	10 891	34 646	218
Colinus virginianus	0	873	873	0	7356	-
Columba livia	1236	1734	498	12 223	33 904	177
Contopus cooperi	1545	1245	**-300**	18 679	40 416	116
Contopus virens	1235	1744	509	12 446	36 639	194
Corvus brachyrhynchos	1536	1749	213	18 832	38 036	102
Cyanocitta cristata	1458	1748	290	16 085	37 749	135
Dendroica caerulescens	1508	1501	**-7**	17 644	40 903	132
Dendroica castanea	1519	998	**-521**	19 057	37 102	95
Dendroica cerulea	0	857	857	0	8260	-
Dendroica discolor	33	1055	1022	101	10 394	10 176
Dendroica dominica	0	174	174	0	405	-
Dendroica fusca	1513	1553	40	17 696	41 952	137
Dendroica magnolia	1603	1434	**-169**	21 597	41 768	93

Species	Number of protected areas located in the species distribution		Potential change in number of protected areas	Protected range (km^2)		Potential change in protected range
	Modelled distribution 1961-1990	Modeled distribution 2071-2100		Modelled distribution 1961-1990	Modelled distribution 2071-2100	
Dendroica palmarum	1209	791	**-418**	14 362	32 646	127
Dendroica pensylvanica	1541	1749	208	18 342	42 339	131
Dendroica petechia	1497	1749	252	17 786	37 928	113
Dendroica pinus	751	1657	906	4206	25 069	496
Dendroica tigrina	1467	1045	**-422**	18 691	38 166	104
Dendroica virens	1537	1749	212	19 657	42 583	117
Dolichonyx oryzivorus	1428	1745	317	15 995	36 687	129
Dryocopus pileatus	1379	1608	229	14 492	34 148	136
Dumetella carolinensis	1419	1748	329	15 158	37 865	150
Empidonax alnorum	1602	1748	146	22 496	42 581	89
Empidonax flaviventris	1573	1076	**-497**	19 761	38 807	96
Empidonax minimus	1557	1748	191	19 619	41 596	112
Empidonax traillii	564	1683	1119	1865	24 950	1238
Empidonax virescens	0	755	755	0	5379	-
Euphagus carolinus	1490	983	**-507**	21 448	37 538	75
Falco sparverius	1517	1749	232	18 027	37 741	109
Fulica americana	424	252	**-172**	1793	3526	97
Gallinula chloropus	572	569	**-3**	1699	6895	306
Geothlypis trichas	1578	1749	171	19 877	42 583	114
Haliaeetus leucocephalus	594	448	**-146**	4948	16 789	239
Helmitheros vermivorum	21	803	782	906	7414	718
Hirundo rustica	1545	1749	204	19 423	42 339	118
Hylocichla mustelina	1171	1735	564	11 997	33 837	182

Species	Number of protected areas located in the species distribution		Potential change in number of protected areas	Protected range (km^2)		Potential change in protected range
	Modelled distribution 1961-1990	Modeled distribution 2071-2100		Modelled distribution 1961-1990	Modelled distribution 2071-2100	
Icteria virens	5	797	792	184	5762	3027
Icterus galbula	1041	1721	680	9894	31 571	219
Icterus spurius	0	980	980	0	9091	-
Ixobrychus exilis	480	253	**-227**	1340	3607	169
Lanius ludovicianus	386	112	**-274**	2262	2301	2
Larus delawarensis	1404	1745	341	17 110	39 375	130
Lophodytes cucullatus	1245	1223	-22	11 647	35 877	208
Loxia curvirostra	1104	1273	169	11 165	40 434	262
Megaceryle alcyon	1557	1749	192	19 090	40 535	112
Megascops asio	6	965	959	17	8656	50 463
Melanerpes carolinus	0	766	766	0	5071	-
Melanerpes erythrocephalus	375	1414	1039	1499	15 580	939
Meleagris gallopavo	0	260	260	0	4238	-
Melospiza georgiana	1537	1749	212	19 846	42 583	115
Melospiza melodia	1537	1749	212	19 526	40 676	108
Mimus polyglottos	1031	1635	604	8344	25 652	207
Mniotilta varia	1511	1733	222	18 212	39 652	118
Molothrus ater	1483	1749	266	17 991	37 741	110
Myiarchus crinitus	972	1717	745	8658	31 087	259
Nycticorax nycticorax	849	1588	739	6395	32 105	402
Oporornis agilis	269	217	**-52**	5241	13 295	154
Oporornis formosus	0	545	545	0	2586	-
Oporornis philadelphia	1582	1526	-56	19 764	41 982	112

Species	Number of protected areas located in the species distribution		Potential change in number of protected areas	Protected range (km^2)		Potential change in protected range
	Modelled distribution 1961-1990	Modeled distribution 2071-2100		Modelled distribution 1961-1990	Modelled distribution 2071-2100	
Pandion haliaetus	1547	1730	183	21 702	42 388	95
Parula americana	1409	1631	222	16 172	39 371	143
Passer domesticus	1416	1747	331	15 233	37 085	143
Passerina caerulea	0	206	206	0	721	-
Passerina cyanea	945	1726	781	7935	33 014	316
Petrochelidon pyrrhonota	1398	1744	346	14 250	36 313	155
Phasianus colchicus	400	1571	1171	1273	17 534	1277
Pheucticus ludovicianus	1491	1749	258	17 506	41 341	136
Picoides arcticus	1295	876	**-419**	17 459	35 320	102
Picoides pubescens	1479	1748	269	16 731	37 949	127
Picoides villosus	1506	1749	243	17 530	38 441	119
Pipilo erythrophthalmus	574	1665	1091	2079	23 319	1022
Piranga olivacea	1323	1734	411	12 713	33 352	162
Piranga rubra	12	245	233	527	1401	166
Plegadis falcinellus	130	355	225	461	1913	315
Podiceps grisegena	432	130	-302	3377	11 688	246
Podilymbus podiceps	1252	1537	285	12 111	35 479	193
Poecile atricapillus	1508	1749	241	18 117	38 676	113
Poecile carolinensis	0	241	241	0	801	-
Polioptila caerulea	98	1496	1398	1047	17 140	1536
Pooecetes gramineus	1313	1733	420	12 505	33 625	169
Porzana carolina	1012	1481	469	7124	34 645	386
Progne subis	720	1698	978	3427	27 871	713

Species	Number of protected areas located in the species distribution		Potential change in number of protected areas	Protected range (km^2)		Potential change in protected range
	Modelled distribution 1961-1990	Modeled distribution 2071-2100		Modelled distribution 1961-1990	Modelled distribution 2071-2100	
Protonotaria citrea	0	333	333	0	847	-
Quiscalus quiscula	1539	1749	210	19 105	40 590	112
Rallus limicola	770	1431	661	3590	27 464	665
Regulus satrapa	1546	1375	**-171**	20 201	41 461	105
Riparia riparia	1507	1749	242	18 583	40 386	117
Sayornis phoebe	1219	1732	513	11 708	33 758	188
Scolopax minor	1316	1743	427	13 983	37 335	167
Seiurus aurocapilla	1505	1747	242	17 371	39 769	129
Seiurus motacilla	12	919	907	55	8803	15 810
Setophaga ruticilla	1549	1749	200	19 223	42 325	120
Sialia sialis	886	1733	847	5571	33 253	497
Sitta canadensis	1541	1653	112	19 054	42 315	122
Sitta carolinensis	1047	1719	672	9765	30 992	217
Sphyrapicus varius	1545	1539	**-6**	19 055	42 035	121
Spiza americana	0	183	183	0	695	-
Spizella pallida	587	717	130	2593	19 429	649
Spizella passerina	1492	1749	257	17 802	37 834	113
Spizella pusilla	688	1697	1009	3112	26 262	744
Stelgidopteryx serripennis	629	1686	1057	2810	25 189	796
Strix varia	843	1684	841	6974	33 639	382
Sturnella magna	1069	1726	657	10 230	32 785	220
Sturnella neglecta	378	324	**-54**	1283	5803	352
Tachycineta bicolor	1582	1749	167	20 822	42 583	105

Species	Number of protected areas located in the species distribution		Potential change in number of protected areas	Protected range (km^2)		Potential change in protected range
	Modelled distribution 1961-1990	Modeled distribution 2071-2100		Modelled distribution 1961-1990	Modelled distribution 2071-2100	
Thryothorus ludovicianus	0	823	823	0	6879	-
Toxostoma rufum	1144	1728	584	10 400	32 905	216
Tringa flavipes	808	332	**-476**	6333	16 724	164
Tringa melanoleuca	932	468	**-464**	10 294	23 038	124
Tringa solitaria	1283	537	**-746**	17 014	27 155	60
Troglodytes aedon	832	1716	884	4924	32 153	553
Troglodytes troglodytes	1600	1544	**-56**	22 622	42 050	86
Tyrannus tyrannus	1504	1749	245	17 765	38 550	117
Vermivora celata	358	318	**-40**	3440	24 430	610
Vermivora chrysoptera	474	1533	1059	1630	18 690	1047
Vermivora peregrina	1556	1021	**-535**	20 911	38 140	82
Vermivora pinus	0	1180	1180	0	12 134	-
Vermivora ruficapilla	1586	1551	**-35**	20 174	42 067	109
Vireo flavifrons	491	1636	1145	1186	21 255	1693
Vireo gilvus	1076	1718	642	10 184	32 061	215
Vireo griseus	0	639	639	0	3015	-
Vireo olivaceus	1562	1749	187	18 415	42 583	131
Vireo philadelphicus	1551	896	**-655**	20 793	35 730	72
Vireo solitarius	1574	1530	**-44**	18 920	41 441	119
Wilsonia canadensis	1515	1551	36	18 625	41 823	125
Wilsonia citrina	0	827	827	0	7382	-
Zenaida macroura	1268	1746	478	12 587	37 621	199
Zonotrichia albicollis	1626	1486	**-140**	23 478	41 916	79

Species	Number of protected areas located in the species distribution		Potential change in number of protected areas	Protected range (km²)		Potential change in protected range
	Modelled distribution 1961-1990	Modeled distribution 2071-2100		Modelled distribution 1961-1990	Modelled distribution 2071-2100	
Amphibians (n = 40)						
Acris crepitans	0	0	0	0	0	-
Ambystoma barbouri	0	96	96	0	139	-
Ambystoma jeffersonianum	38	1028	990	68	10 155	14 743
Ambystoma laterale	1132	1413	281	10 159	31 524	210
Ambystoma maculatum	935	1600	665	7513	20 837	177
Ambystoma opacum	0	521	521	0	1770	-
Ambystoma texanum	0	25	25	0	45	-
Ambystoma tigrinum mavortium complex	0	181	181	0	454	-
Anaxyrus americanus	0	515	515	0	1930	-
Anaxyrus fowleri	0	32	32	0	325	-
Aneides aeneus	1650	1666	16	21 994	40 493	84
Cryptobranchus alleganiensis	0	224	224	0	2654	-
Desmognathus fuscus	623	1497	874	2530	18 612	636
Desmognathus ochrophaeus	207	1339	1132	527	13 888	2535
Eurycea bislineata	1152	1615	463	12 434	25 990	109
Eurycea longicauda	0	317	317	0	4252	-
Gyrinophilus porphyriticus	261	1060	799	787	14 467	1738
Hemidactylium scutatum	12	1054	1042	45	10 562	23 283
Hyla chrysoscelis	0	14	14	0	37	-
Hyla versicolor	649	1463	814	3193	16 943	431
Lithobates catesbeianus	769	1579	810	4949	18 465	273

Species	Number of protected areas located in the species distribution		Potential change in number of protected areas	Protected range (km^2)		Potential change in protected range
	Modelled distribution 1961-1990	Modeled distribution 2071-2100		Modelled distribution 1961-1990	Modelled distribution 2071-2100	
Lithobates clamitans	1207	1671	464	12 351	25 764	109
Lithobates heckscheri	0	0	0	0	0	-
Lithobates palustris	805	1578	773	5335	19 735	270
Lithobates pipiens	1110	1708	598	10 024	31 170	211
Lithobates septentrionalis	1619	1086	**-533**	22 008	38 714	76
Lithobates sphenocephalus	0	143	143	0	374	-
Lithobates sylvaticus	1512	1680	168	19 251	35 299	83
Necturus maculosus	193	1000	807	571	8419	1375
Notophthalmus viridescens	948	1588	640	7268	21 532	196
Plethodon cinereus	977	1655	678	7948	24 594	209
Plethodon electromorphus	0	379	379	0	1089	-
Plethodon glutinosus	0	932	932	0	7593	-
Plethodon hoffmani	0	78	78	0	1130	-
Plethodon wehrlei	0	124	124	0	1752	-
Pseudacris brachyphona	0	4	4	0	171	-
Pseudacris crucifer	1225	1603	378	13 148	25 033	90
Pseudacris triseriata maculata complex	4	155	151	6	642	10 751
Pseudotriton ruber	0	160	160	0	2 204	-
Scaphiopus holbrookii	0	72	72	0	101	-
Trees (n = 90)						
Abies balsamea	1731	888	**-843**	37 035	33 559	**-9**
Acer negundo	0	9	9	0	18	-

Species	Number of protected areas located in the species distribution		Potential change in number of protected areas	Protected range (km^2)		Potential change in protected range
	Modelled distribution 1961-1990	Modeled distribution 2071-2100		Modelled distribution 1961-1990	Modelled distribution 2071-2100	
Acer nigrum	233	824	591	487	8691	1684
Acer pensylvanicum	1240	1351	111	13 106	33 762	158
Acer rubrum	1276	1706	430	12 327	31 556	156
Acer saccharinum	288	615	327	763	2762	262
Acer saccharum	1055	1382	327	10 457	28 908	176
Aesculus glabra	0	50	50	0	518	-
Aesculus octandra	0	32	32	0	549	-
Alnus glutinosa	9	811	802	35	11 644	32 858
Asimina triloba	0	9	9	0	19	-
Betula alleghaniensis	1352	1437	85	15 416	39 287	155
Betula lenta	0	985	985	0	13 516	-
Betula nigra	0	146	146	0	334	-
Betula papyrifera	1713	1133	**-580**	33 068	37 298	13
Betula populifolia	769	1688	919	3808	32 994	767
Carya aquatica	41	473	432	159	3152	1884
Carya cordiformis	80	537	457	201	3689	1735
Carya glabra	0	227	227	0	616	-
Carya laciniosa	0	23	23	0	79	-
Carya ovata	66	814	748	180	5742	3084
Carya tomentosa	0	132	132	0	341	-
Castanea dentata	0	360	360	0	1980	-
Catalpa speciosa	0	177	177	0	986	-
Celtis occidentalis	0	609	609	0	3610	-

Species	Number of protected areas located in the species distribution		Potential change in number of protected areas	Protected range (km^2)		Potential change in protected range
	Modelled distribution 1961-1990	Modeled distribution 2071-2100		Modelled distribution 1961-1990	Modelled distribution 2071-2100	
Cercis canadensis	0	19	19	0	73	-
Chamaecyparis thyoides	41	470	429	59	1782	2904
Cornus florida	0	73	73	0	156	-
Fagus grandifolia	741	1453	712	5225	21 624	314
Fraxinus americana	574	1508	934	2277	20 096	783
Fraxinus nigra	1165	1540	375	11 025	34 545	213
Fraxinus pennsylvanica	298	639	341	777	2781	258
Gleditsia triacanthos	0	59	59	0	197	-
Gymnocladus dioicus	0	4	4	0	4	-
Ilex opaca	0	56	56	0	120	-
Juglans cinerea	496	1527	1031	1754	19 758	1026
Juglans nigra	0	21	21	0	53	-
Juniperus virginiana	0	216	216	0	806	-
Larix laricina	1648	812	**-836**	33 990	28 864	**-15**
Liquidambar styraciflua	0	242	242	0	594	-
Liriodendron tulipifera	0	473	473	0	1553	-
Maclura pomifera	19	279	260	294	4699	1497
Magnolia acuminata	0	23	23	0	841	-
Magnolia grandiflora	5	11	6	29	99	242
Magnolia macrophylla	0	209	209	0	831	-
Magnolia virginiana	0	318	318	0	815	-
Morus rubra	0	9	9	0	20	-
Nyssa sylvatica	0	295	295	0	726	-

Species	Number of protected areas located in the species distribution		Potential change in number of protected areas	Protected range (km^2)		Potential change in protected range
	Modelled distribution 1961-1990	Modeled distribution 2071-2100		Modelled distribution 1961-1990	Modelled distribution 2071-2100	
Ostrya virginiana	691	1393	702	4035	21 229	426
Picea glauca	990	247	**-743**	29 853	17 836	**-40**
Picea mariana	1615	898	**-717**	22 592	33 465	48
Picea rubens	1664	932	**-732**	39 855	34 384	**-14**
Pinus banksiana	4	14	10	6	327	5045
Pinus pungens	700	1191	491	7572	29 104	284
Pinus resinosa	0	1062	1062	0	9283	-
Pinus rigida	1252	1325	73	14 372	36 607	155
Pinus strobus	1190	1494	304	11 393	33 327	193
Pinus virginiana	0	46	46	0	104	-
Platanus occidentallis	0	101	101	0	261	-
Populus balsamifera	1212	1579	367	12 304	32 922	168
Populus deltoides	338	639	301	910	2958	225
Populus grandidentata	937	1539	602	8657	29 180	237
Populus tremuloides	1652	1309	**-343**	23 721	38 231	61
Prunus americana	0	0	0	0	0	-
Prunus pensylvanica	1651	1517	**-134**	23 643	39 450	67
Prunus serotina	93	1005	912	174	12 570	7137
Quercus alba	0	780	780	0	5637	-
Quercus bicolor	253	946	693	674	7411	1000
Quercus coccinea	0	831	831	0	5777	-
Quercus ellipsoidalis	4	17	13	5	32	535
Quercus ilicifolia	0	959	959	0	7955	-

Species	Number of protected areas located in the species distribution		Potential change in number of protected areas	Protected range (km^2)		Potential change in protected range
	Modelled distribution 1961-1990	Modeled distribution 2071-2100		Modelled distribution 1961-1990	Modelled distribution 2071-2100	
Quercus imbricaria	0	45	45	0	116	-
Quercus macrocarpa	291	487	196	890	3854	333
Quercus muehlenbergii	0	22	22	0	44	-
Quercus palustris	0	555	555	0	1914	-
Quercus prinus	0	388	388	0	3603	-
Quercus rubra	201	1177	976	1858	15 470	732
Quercus velutina	0	407	407	0	1921	-
Robinia pseudoacacia	0	394	394	0	2148	-
Salix nigra	6	145	139	17	442	2483
Sassafras albidum	0	395	395	0	1101	-
Sorbus americana	1727	1464	**-263**	32 732	38 755	18
Taxodium distichum	0	0	0	0	0	-
Thuja occidentalis	1386	1466	80	14 148	38 060	169
Tilia americana	646	1430	784	2918	22 462	670
Tilia heterophylla	2	103	101	14	1185	8465
Tsuga canadensis	762	1545	783	5089	33 290	554
Ulmus americana	591	1529	938	2707	18 540	585
Ulmus rubra	34	528	494	130	3575	2655
Ulmus thomasii	382	953	571	1316	10 711	714
Other vascular plants (n = 223)						
Achillea millefolium	517	1404	887	1485	14 650	887
Actaea pachypoda	560	1530	970	1939	17 433	799

Species	Number of protected areas located in the species distribution		Potential change in number of protected areas	Protected range (km^2)		Potential change in protected range
	Modelled distribution 1961-1990	Modeled distribution 2071-2100		Modelled distribution 1961-1990	Modelled distribution 2071-2100	
Actaea rubra	1552	1123	**-429**	17 739	38 476	117
Adiantum pedatum	248	1259	1011	617	11 984	1843
Ageratina altissima var. altissima	282	1308	1026	754	12 667	1581
Alectoria ochroleuca	7	0	**-7**	4812	0	**-100**
Allium tricoccum	614	1483	869	2331	17 104	634
Anaphalis margaritacea	1530	1306	**-224**	17 582	39 559	125
Andromeda polifolia	740	347	**-393**	17 129	5318	**-69**
Anemone acutiloba	354	960	606	913	8194	798
Anemone canadensis	464	992	528	1500	12 910	761
Apocynum androsaemifolium	986	1479	493	8508	26 018	206
Aralia hispida	940	1293	353	7236	27 326	278
Aralia nudicaulis	1648	1369	**-279**	24 513	41 388	69
Aralia racemosa	582	1511	929	2344	16 177	590
Arctostaphylos uva-ursi	514	1080	566	2085	14 323	587
Arctous alpina	67	0	**-67**	8893	0	**-100**
Arisaema triphyllum	687	1608	921	3679	21 722	490
Aronia melanocarpa	631	1538	907	2754	19 660	614
Asarum canadense	300	1194	894	806	11 242	1294
Asclepias syriaca	273	793	520	738	6212	742
Athyrium filix-femina	1513	1673	160	17 379	41 721	140
Betula glandulosa	130	0	**-130**	20 036	0	**-100**
Betula minor	172	0	**-172**	23 545	0	**-100**
Betula pumila	419	56	**-363**	25 999	1334	**-95**

Species	Number of protected areas located in the species distribution		Potential change in number of protected areas	Protected range (km²)		Potential change in protected range
	Modelled distribution 1961-1990	Modeled distribution 2071-2100		Modelled distribution 1961-1990	Modelled distribution 2071-2100	
Botrychium virginianum	662	1478	816	3884	16 876	334
Calypso bulbosa	1166	400	**-766**	11 087	15 874	43
Capnoides sempervirens	546	1257	711	1720	16 076	835
Cardamine diphylla	619	1431	812	2462	19 270	683
Caulophyllum thalictroides	513	1484	971	1766	15 408	773
Chamerion angustifolium subsp. angustifolium	1333	162	**-1171**	34 106	23 477	**-31**
Chimaphila umbellata	648	1188	540	3478	13 388	285
Circaea alpina	1527	1360	**-167**	17 475	36 888	111
Circaea lutetiana	275	923	648	752	7645	916
Cladonia crispata	98	0	**-98**	13 284	0	**-100**
Cladonia uncialis	67	0	**-67**	13 930	0	**-100**
Claytonia caroliniana	643	1218	575	2830	15 943	463
Clintonia borealis	1700	740	**-960**	40 613	32 805	**-19**
Comarum palustre	641	980	339	2921	15 995	448
Comptonia peregrina	498	1363	865	2353	15 344	552
Coptis trifolia	1716	778	**-938**	42 173	34 099	**-19**
Corallorhiza maculata	683	1286	603	3359	19 812	490
Cornus alternifolia	1195	1671	476	11 435	31 168	173
Cornus canadensis	1596	334	**-1262**	42 107	27 396	**-35**
Cornus stolonifera	1556	1170	**-386**	17 889	39 744	122
Corylus cornuta	1531	1392	**-139**	16 636	40 515	144
Cypripedium acaule	1459	1622	163	14 835	34 667	134
Cypripedium parviflorum var. pubescens	305	1413	1108	658	14 345	2079

Species	Number of protected areas located in the species distribution		Potential change in number of protected areas	Protected range (km^2)		Potential change in protected range
	Modelled distribution 1961-1990	Modeled distribution 2071-2100		Modelled distribution 1961-1990	Modelled distribution 2071-2100	
Cypripedium reginae	676	1142	466	3028	17 551	480
Cystopteris bulbifera	284	1323	1039	651	12 783	1863
Dasiphora fruticosa	313	1019	706	842	13 165	1463
Dennstaedtia punctilobula	733	1527	794	4475	22 199	396
Deparia acrostichoides	448	1426	978	1254	15 987	1175
Diapensia lapponica	283	32	**-251**	17 454	5918	**-66**
Dicentra canadensis	310	980	670	830	8422	915
Dicentra cucullaria	286	926	640	797	7118	793
Diervilla lonicera	1596	1272	**-324**	20 096	40 357	101
Diphasiastrum ×sabinifolium	90	0	**-90**	18 969	0	**-100**
Diphasiastrum complanatum	1659	513	**-1146**	27 367	29 641	8
Diphasiastrum digitatum	374	1383	1009	921	14 688	1494
Diphasiastrum tristachyum	558	1532	974	1961	17 787	807
Doellingeria umbellata var. umbellata	466	1405	939	1316	17 222	1209
Dryopteris cristata	923	1653	730	7021	29 941	326
Dryopteris goldiana	354	1343	989	906	13 813	1425
Dryopteris intermedia	1690	1390	**-300**	32 260	41 294	28
Dryopteris marginalis	659	1512	853	3310	17 458	427
Echinocystis lobata	460	1176	716	1098	13 061	1090
Empetrum nigrum	94	0	**-94**	14 230	0	**-100**
Epifagus virginiana	230	1017	787	555	9672	1643
Epigaea repens	1388	1396	8	24 551	28 052	14
Epilobium palustre	1393	428	**-965**	20 026	22 526	12

Species	Number of protected areas located in the species distribution		Potential change in number of protected areas	Protected range (km^2)		Potential change in protected range
	Modelled distribution 1961-1990	Modeled distribution 2071-2100		Modelled distribution 1961-1990	Modelled distribution 2071-2100	
Epipactis helleborine	636	1236	600	2596	18 308	605
Equisetum sylvaticum	1157	394	**-763**	31 795	19 921	**-37**
Erigeron canadensis	0	518	518	0	2985	-
Erythronium americanum	338	1366	1028	767	13 185	1618
Eurybia macrophylla	1552	1398	**-154**	17 693	37 607	113
Eutrochium maculatum var. maculatus	827	1316	489	5469	21 785	298
Fallopia cilinodis	857	1293	436	5807	24 653	325
Flavocetraria nivalis	10	0	**-10**	6570	0	**-100**
Galium asprellum	541	1220	679	1706	15 329	799
Galium labradoricum	1276	739	**-537**	11 869	21 803	84
Galium triflorum	843	1337	494	7558	13 653	81
Gaultheria hispidula	1345	197	**-1148**	40 766	24 324	**-40**
Gaultheria procumbens	1074	1562	488	9333	24 328	161
Gaylussacia baccata	46	880	834	109	6803	6161
Geocaulon lividum	491	0	**-491**	29 550	0	**-100**
Geum macrophyllum	1514	439	**-1075**	18 160	28 892	59
Geum rivale	1134	1144	10	10 093	34 311	240
Goodyera repens	1553	544	**-1009**	25 509	30 117	18
Gymnocarpium dryopteris	1707	997	**-710**	37 330	37 293	0
Heracleum maximum	530	1426	896	2583	19 653	661
Hieracium vulgatum	816	1059	243	10 149	23 779	134
Huperzia lucidula	1561	1659	98	19 552	41 885	114
Impatiens capensis	832	1623	791	6101	21 221	248

Species	Number of protected areas located in the species distribution		Potential change in number of protected areas	Protected range (km^2)		Potential change in protected range
	Modelled distribution 1961-1990	Modeled distribution 2071-2100		Modelled distribution 1961-1990	Modelled distribution 2071-2100	
Juniperus communis	131	615	484	2645	6277	137
Juniperus horizontalis	588	441	**-147**	8339	11 495	38
Kalmia angustifolia	1479	225	**-1254**	35 824	24 742	**-31**
Kalmia procumbens	93	0	**-93**	13 570	0	**-100**
Laportea canadensis	462	1437	975	1285	14 621	1038
Larix decidua	1749	1749	0	42 583	42 583	0
Leucanthemum vulgare	198	1042	844	514	9458	1741
Linnaea borealis	1483	255	**-1228**	40 122	25 709	**-36**
Listera cordata	604	91	**-513**	31 489	14 494	**-54**
Lonicera canadensis	1474	1070	**-404**	15 477	32 923	113
Lonicera hirsuta	584	156	**-428**	2489	2764	11
Lonicera villosa	655	85	**-570**	25 219	10 271	**-59**
Lycopodium annotinum	1446	354	**-1092**	41 561	27 823	**-33**
Lycopodium clavatum	1632	1337	**-295**	24 096	40 550	68
Lycopodium obscurum	1690	1360	**-330**	30 092	40 463	34
Lycopodium selago	588	675	87	15 067	24 553	63
Lycopus americanus	326	1094	768	839	9873	1077
Maianthemum canadense	1742	1173	**-569**	36 515	39 670	9
Maianthemum racemosum subsp. racemosum	1004	1639	635	9189	24 164	163
Maianthemum stellatum	565	1082	517	2267	13 912	514
Maianthemum trifolium	1234	117	**-1117**	38 903	18 857	**-52**
Matteuccia struthiopteris	1044	1371	327	8318	33 669	305
Medeola virginiana	961	1637	676	8803	28 653	226

Species	Number of protected areas located in the species distribution		Potential change in number of protected areas	Protected range (km²)		Potential change in protected range
	Modelled distribution 1961-1990	Modeled distribution 2071-2100		Modelled distribution 1961-1990	Modelled distribution 2071-2100	
Melampyrum lineare	1297	1326	29	13 486	25 300	88
Mentha arvensis subsp. Borealis	508	1375	867	1318	14 504	1000
Mertensia paniculata	245	9	**-236**	4323	66	**-98**
Mitchella repens	735	1579	844	4697	20 312	332
Mitella diphylla	291	1061	770	798	9186	1051
Mitella nuda	1610	378	**-1232**	27 105	28 411	5
Moneses uniflora	1372	372	**-1000**	22 731	27 498	21
Monotropa hypopithys	14	871	857	23	5718	24 388
Monotropa uniflora	1490	1712	222	18 171	42 037	131
Myrica gale	1293	719	**-574**	15 048	26 938	79
Oclemena acuminata	1476	1289	**-187**	16 150	36 850	128
Onoclea sensibilis	827	1588	761	6381	21 279	233
Orthilia secunda	1643	650	**-993**	31 277	30 094	**-4**
Osmorhiza claytonii	564	1505	941	1965	15 891	709
Osmunda claytoniana	1550	1650	100	20 479	41 626	103
Osmunda regalis	456	1322	866	1247	14 238	1042
Osmundastrum cinnamomeum	1324	1594	270	13 529	35 884	165
Oxalis dillenii	213	746	533	525	4854	825
Oxalis montana	1600	1178	**-422**	23 180	38 482	66
Parthenocissus quinquefolia	273	868	595	737	6808	823
Petasites frigidus var. palmatus	931	52	**-879**	35 157	12 076	**-66**
Phegopteris connectilis	1597	1178	**-419**	24 317	39 420	62
Plantago major	521	1269	748	1640	15 252	830

Species	Number of protected areas located in the species distribution		Potential change in number of protected areas	Protected range (km^2)		Potential change in protected range
	Modelled distribution 1961-1990	Modeled distribution 2071-2100		Modelled distribution 1961-1990	Modelled distribution 2071-2100	
Platanthera orbiculata	1170	1276	106	10 393	24 438	135
Polygonatum pubescens	1001	1506	505	9157	25 923	183
Polypodium sibiricum	1215	1685	470	11 604	32 034	176
Polystichum acrostichoides	321	1296	975	687	12 698	1749
Polystichum braunii	1094	611	**-483**	12 782	27 122	112
Potentilla norvegica	290	1157	867	669	10 837	1520
Potentilla simplex	193	866	673	456	6889	1412
Prunella vulgaris	391	1257	866	986	12 090	1127
Pteridium aquilinum	286	1014	728	764	9197	1103
Pyrola asarifolia	1389	213	**-1176**	17 846	18 069	1
Pyrola elliptica	1453	1269	**-184**	14 454	30 387	110
Racomitrium lanuginosum	25	0	**-25**	8113	0	**-100**
Ranunculus abortivus	32	785	753	124	5215	4102
Ranunculus acris	902	1503	601	6566	25 297	285
Rhamnus alnifolia	687	1036	349	3499	18 442	427
Rhamnus cathartica	429	1150	721	1051	13 429	1178
Rhododendron canadense	669	558	**-111**	12 554	15 988	27
Ribes americanum	421	1061	640	1031	12 863	1147
Ribes cynosbati	425	1120	695	1618	12 345	663
Ribes glandulosum	1675	759	**-916**	26 461	33 684	27
Ribes hirtellum	582	1128	546	2513	14 496	477
Ribes lacustre	1609	486	**-1123**	25 152	29 335	17
Ribes triste	1575	408	**-1167**	23 675	28 705	21

Species	Number of protected areas located in the species distribution		Potential change in number of protected areas	Protected range (km^2)		Potential change in protected range
	Modelled distribution 1961-1990	Modeled distribution 2071-2100		Modelled distribution 1961-1990	Modelled distribution 2071-2100	
Rosa acicularis	622	113	**-509**	11 436	2121	**-81**
Rubus allegheniensis	611	1528	917	2310	16 220	602
Rubus chamaemorus	420	1	**-419**	28 888	391	**-99**
Rubus idaeus	1668	1134	**-534**	24 221	39 890	65
Rubus occidentalis	301	978	677	771	8245	969
Rubus odoratus	457	1303	846	1299	14 199	993
Rubus pubescens	1679	959	**-720**	27 248	37 246	37
Rubus repens	1049	1084	35	8804	29 477	235
Sambucus canadensis	325	1266	941	822	12 253	1390
Sambucus racemosa subsp. pubens var. pubens	1573	1327	**-246**	18 495	40 855	121
Sanguinaria canadensis	150	823	673	386	5630	1358
Sanguisorba canadensis	680	1450	770	13 209	28 361	115
Scutellaria galericulata	562	1125	563	2075	14 719	609
Scutellaria lateriflora	435	1320	885	1036	13 184	1173
Shepherdia canadensis	373	37	**-336**	2259	988	**-56**
Sibbaldia tridentata	253	409	156	6302	6958	10
Solidago flexicaulis	417	1448	1031	1048	15 960	1423
Solidago hispida	97	450	353	695	4783	588
Solidago macrophylla	970	99	**-871**	35 438	18 556	**-48**
Solidago rugosa	711	1540	829	4429	21 629	388
Sorbus decora	1410	204	**-1206**	26 196	24 135	**-8**
Spiraea latifolia	1137	1411	274	10 677	31 997	200

Species	Number of protected areas located in the species distribution		Potential change in number of protected areas	Protected range (km^2)		Potential change in protected range
	Modelled distribution 1961-1990	Modeled distribution 2071-2100		Modelled distribution 1961-1990	Modelled distribution 2071-2100	
Spiraea tomentosa	475	1365	890	1210	14 302	1082
Streptopus amplexifolius	1373	426	**-947**	31 145	28 374	**-9**
Streptopus lanceolatus var. lanceolatus	1525	404	**-1121**	25 205	28 186	12
Symphyotrichum puniceum	726	1659	933	4145	24 401	489
Taraxacum officinale	588	1515	927	2274	17 020	648
Taxus canadensis	1522	1256	**-266**	16 314	38 820	138
Thalictrum dioicum	796	1612	816	4994	21 088	322
Thalictrum pubescens	1431	1571	140	15 658	35 658	128
Thelypteris noveboracensis	1191	1602	411	12 407	32 849	165
Tiarella cordifolia	844	1526	682	6243	25 429	307
Toxicodendron radicans var. radicans	230	839	609	561	6637	1083
Trientalis borealis	1743	1022	**-721**	35 858	37 848	6
Trillium cernuum	702	1256	554	4081	17 469	328
Trillium erectum	1291	1456	165	12 992	34 135	163
Trillium grandiflorum	379	1062	683	1104	10 231	827
Trillium undulatum	1375	1289	**-86**	14 928	34 416	131
Tussilago farfara	363	1300	937	822	13 441	1536
Uvularia grandiflora	204	738	534	522	4788	817
Uvularia sessilifolia	486	1397	911	1298	14 986	1054
Vaccinium angustifolium	1680	383	**-1297**	42 361	26 938	**-36**
Vaccinium caespitosum	106	1	**-105**	17 107	1515	**-91**
Vaccinium corymbosum	226	910	684	542	8752	1515
Vaccinium myrtilloides	1559	273	**-1286**	38 697	26 002	**-33**

Species	Number of protected areas located in the species distribution		Potential change in number of protected areas	Protected range (km^2)		Potential change in protected range
	Modelled distribution 1961-1990	Modeled distribution 2071-2100		Modelled distribution 1961-1990	Modelled distribution 2071-2100	
Vaccinium ovalifolium	191	4	**-187**	17 253	3330	**-81**
Vaccinium oxycoccos	489	0	**-489**	27 616	0	**-100**
Vaccinium uliginosum	120	0	**-120**	16 496	0	**-100**
Vaccinium vitis-idaea	197	0	**-197**	20 907	0	**-100**
Veratrum viride	733	1466	733	4791	21 075	340
Veronica officinalis	386	1334	948	1154	13 166	1041
Viburnum edule	971	71	**-900**	30 270	12 664	**-58**
Viburnum lantanoides	1148	1137	**-11**	11 199	27 540	146
Vicia cracca	1387	1436	49	13 076	32 817	151
Viola canadensis	250	869	619	606	7174	1083
Viola macloskeyi	615	1257	642	2639	17 069	547
Viola pubescens var. pubescens	263	1262	999	595	12 178	1947
Vitis labrusca	1	710	709	0	3769	1 712 873
Vitis riparia	329	1033	704	850	9135	974
Waldsteinia fragarioides	281	819	538	681	6393	839
All species combined (n = 529)						
mean	765	1026	261	9605	20 368	4693
AD	595	596	561	10 361	14 365	80 780
median	666	1173	274	5225	19 556	177
MAD	893	688	574	7747	20 429	239

Supplementary Table S2. Number of repetitions for various steps of ecological niche modeling. Modeling was performed on birds, amphibians, trees, and other vascular plants studied to assess the impacts of climate change on the biodiversity of a northern network of protected areas in Quebec, Canada.

	Birds	Amphibians	Trees	Other vascular plants
Split-sample procedures	10	20	10	10
Algorithms	7	7	8	6
Climate change scenarios	7	7	7	4
Models	140	140	80	120
Projections into the future	980	980	560	480

Supplementary Table S3. Definitions and purposes of IUCN protected area categories. Adapted from[24].

Category	Definition	Purpose
Ia	Strict nature reserves	Set aside to protect biodiversity and also possibly geological/geomorphological features, where human visitation, use and impacts are strictly controlled and limited to ensure protection of the conservation values. Can serve as indispensable reference areas for scientific research and monitoring.
II	National parks	Set aside to protect large-scale ecological processes, along with the complement of species and ecosystems characteristic of the area. Also provide a foundation for environmentally and culturally compatible spiritual, scientific, educational, recreational and visitor opportunities. Are large natural or near natural areas.
III	Natural monuments or features	Set aside to protect a specific natural monument, which can be a landform, sea mount, submarine cavern, geological feature such as a cave or even a living feature such as an ancient grove. Are generally small and often have high visitor value.
IV	Habitat/species management areas	Protect particular species or habitats. Management reflects this priority. Many will need regular, active interventions to address the requirements of particular species or to maintain habitats, even though this is not a requirement of the category.
VI	Protected areas with sustainable use of natural resources	Conserve ecosystems and habitats, together with associated cultural values and traditional natural resource management systems. Are generally large, with most of the area in a natural condition, where a proportion is under sustainable natural resource management and where low-level non-industrial use of natural resources compatible with nature conservation is seen as one of the main aims of the area.

Supplementary Table S4. Web links of publicly available sources of data on species, climate, soil, and topography used to assess the impacts of climate change on the biodiversity of a northern network of protected areas in southern Quebec, Canada.

Type of data	Name of database	Source
Biodiversity		
Birds	*Étude des Populations d'Oiseaux du Québec* (ÉPOQ)	http://www.oiseauxqc.org/epoq.jsp
	Breeding Bird Survey (BBS)	http://www.pwrc.usgs.gov/BBS/index.html
Amphibians	*Atlas des Amphibiens et Reptiles du Québec* (AARQ)	http://www.atlasamphibiensreptiles.qc.ca/
	USGS National Amphibian Atlas	https://armi.usgs.gov/national_amphibian_atlas.php
	Atlantic Canada Conservation Data Center (ACCDC)	http://www.accdc.com/
Trees	*MRNF Placettes-échantillons temporaires* (PET; 3rd program)	https://mffp.gouv.qc.ca/forets/inventaire/fiches/donnees-descriptives-pet-poe.jsp
	MRNF Placettes-échantillons permanentes (PEP)	https://mffp.gouv.qc.ca/forets/inventaire/fiches/donnees-descriptives-pet-poe.jsp
	USDA Forest Service Tree Atlas	https://www.fs.fed.us/nrs/atlas/littlefia/index.html
Other vascular plants	USDA Plants Database	https://plants.usda.gov/java/
	MRNF Point d'Observation Écologique	https://mffp.gouv.qc.ca/forets/inventaire/fiches/donnees-descriptives-pet-poe.jsp
Climate		
Future climate	Canadian Regional Climate Model (CRCM4)	http://www.ouranos.ca/
	Global Climate Models made available by the World Climate Research Programme's CMIP phase 3	http://www-pcmdi.llnl.gov/ipcc/about_ipcc.php
Soil and topography		
Soil	Soil Survey Geographic Database (SSURGO; version 2.1, scale 1:24,000)	https://www.nrcs.usda.gov/wps/portal/nrcs/detail/soils/survey/?cid=nrcs142p2_053627
Elevation	Canadian Digital Elevation Data (CDED)	http://www.geogratis.gc.ca/

Supplementary Table S5. Climatic variables used to calibrate ecological niche models for different groups of species.

Climatic variable	Birds	Amphibians	Trees	Other vascular plants
Mean annual temperature	x	x	x	
Temperature annual range[a]	x	x		
Annual growing degree days				x
Total annual precipitation		x	x	x
Useful precipitation[b]			x	
Precipitation of wettest month[c]	x			
Precipitation seasonality[d]	x	x		
Mean annual water balance[e]				x

[a]Difference between 30-year averages of the mean temperature of the warmest month and the mean temperature of the coldest month.

[b]Ratio of summer precipitation to total annual precipitation. This represents the quantity of precipitation during the growth period of trees[25].

[c]Total precipitation in the wettest month.

[d]Coefficient of variation of total annual precipitation.

[e]Difference between total annual precipitation and potential annual evapotranspiration calculated with the Thornthwaite equation.

Supplementary Figure S1. Frequency distribution of potential representativity of 1,749 protected areas of southern Quebec, Canada (analysis at the scale of the study area). The period 1961-1990 is represented by grey bars and 2071-2100 by hatched bars. Representativity is defined as the proportion of species that occur in the study area that also occur in the cells containing a protected area. The frequency distribution of the potential change in potential representativity between 1961-1990 and 2071-2100 is represented by bars with thick borders.

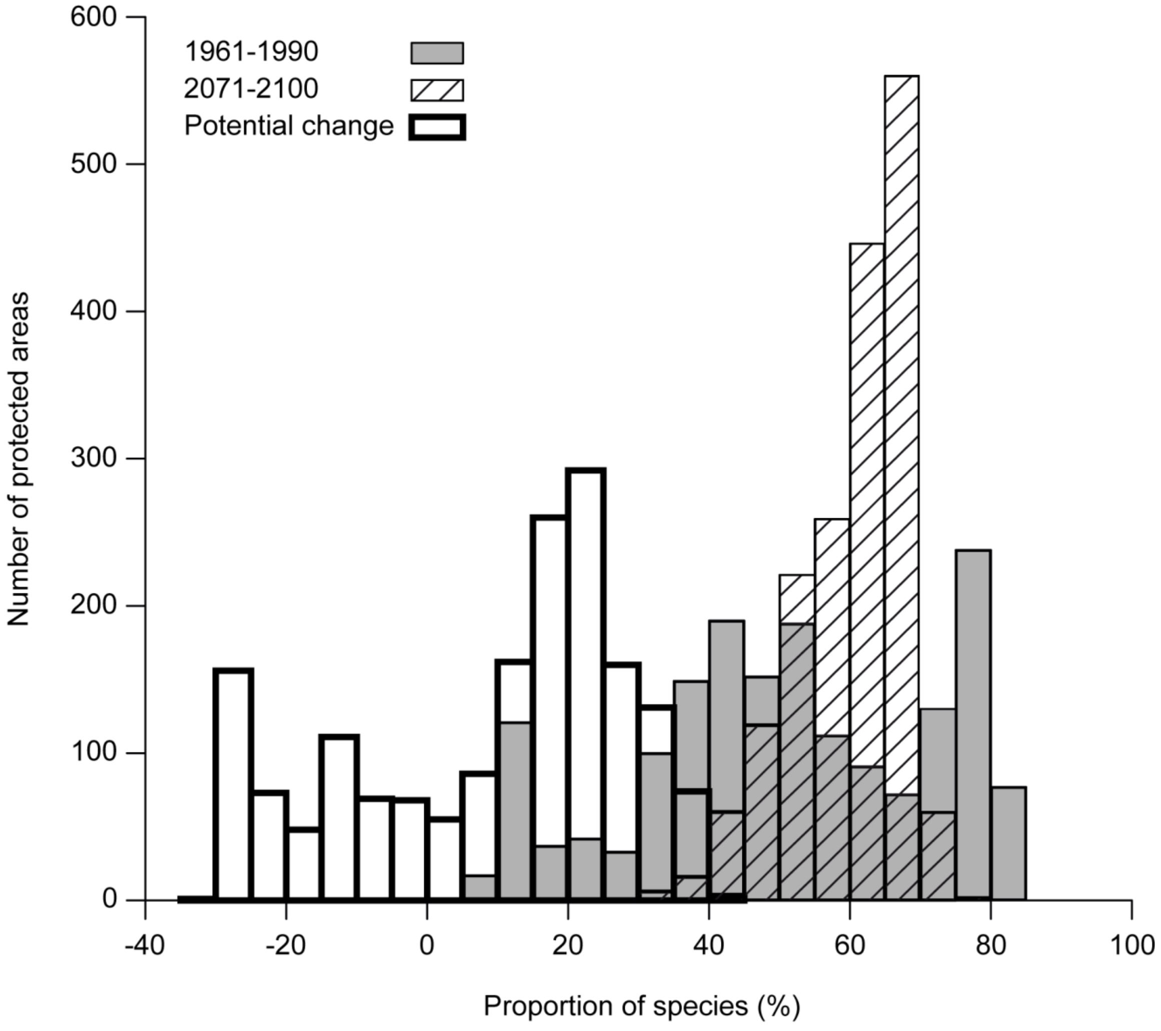

Supplementary Figure S2. Frequency distribution of potential representativity of 1,711 protected areas of southern Quebec, Canada (analysis at the scale of the natural region). The period 1961-1990 is represented by grey bars and 2071-2100 by hatched bars. Representativity is defined as the proportion of species that occur in the cells containing a given natural region (as defined in Quebec's Ecological Reference Framework) that also occur in the cells containing a protected area located in that natural region. The frequency distribution of the potential change in potential representativity between 1961-1990 and 2071-2100 is represented by bars with thick borders.

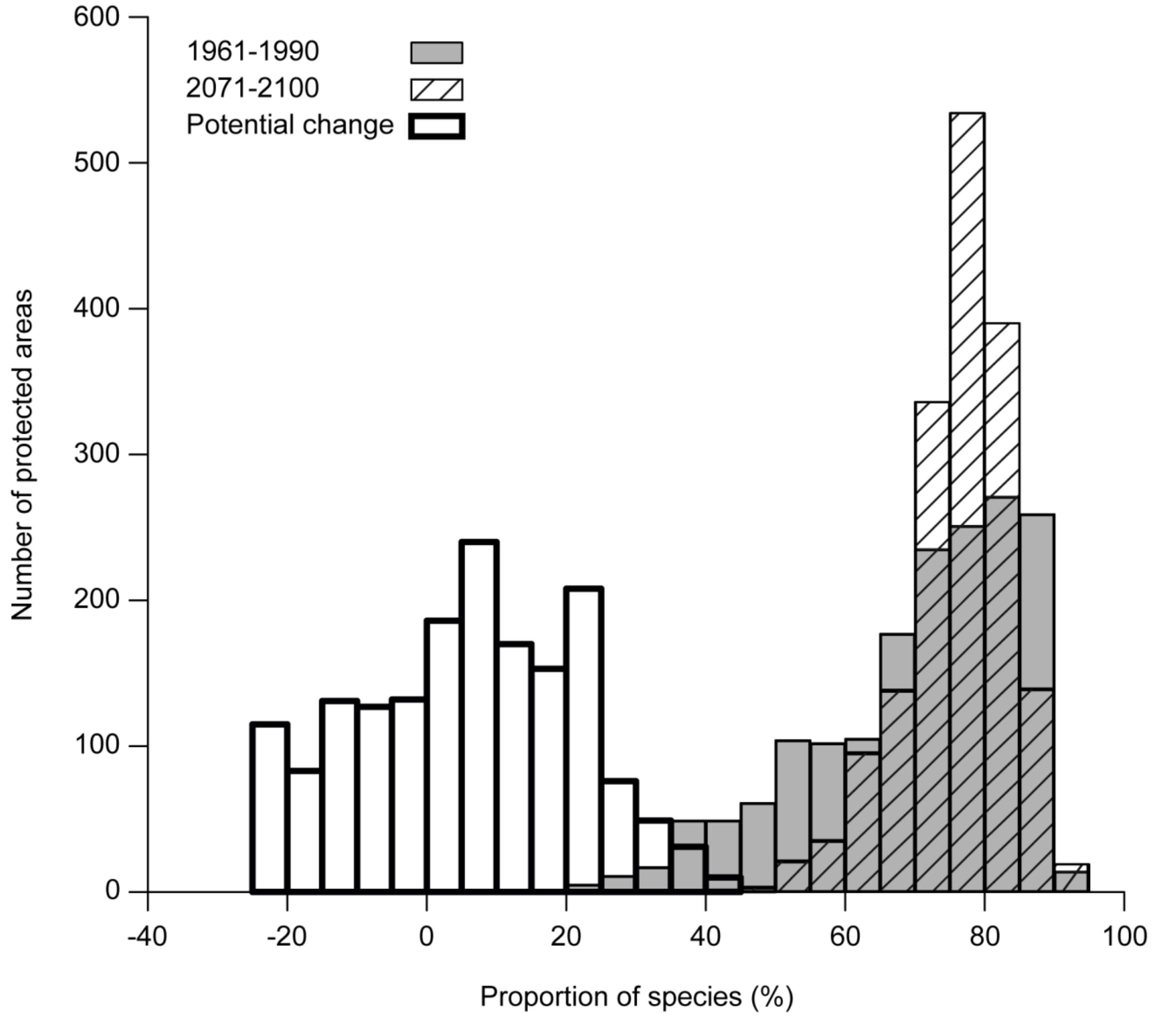

Supplementary Figure S3. Protected areas included in and excluded from the study area located in southern Quebec, Canada. Included protected areas are in blue (n = 1749) and excluded protected areas are in red (n = 690). Ecological niche modelling used to evaluate the potential impacts of climate change on biodiversity was performed on a modeling area (hatching on the top left box) much wider than our Quebec study area. Maps were created using ESRI ArcGIS 9.4 (http://www.esri.com/).

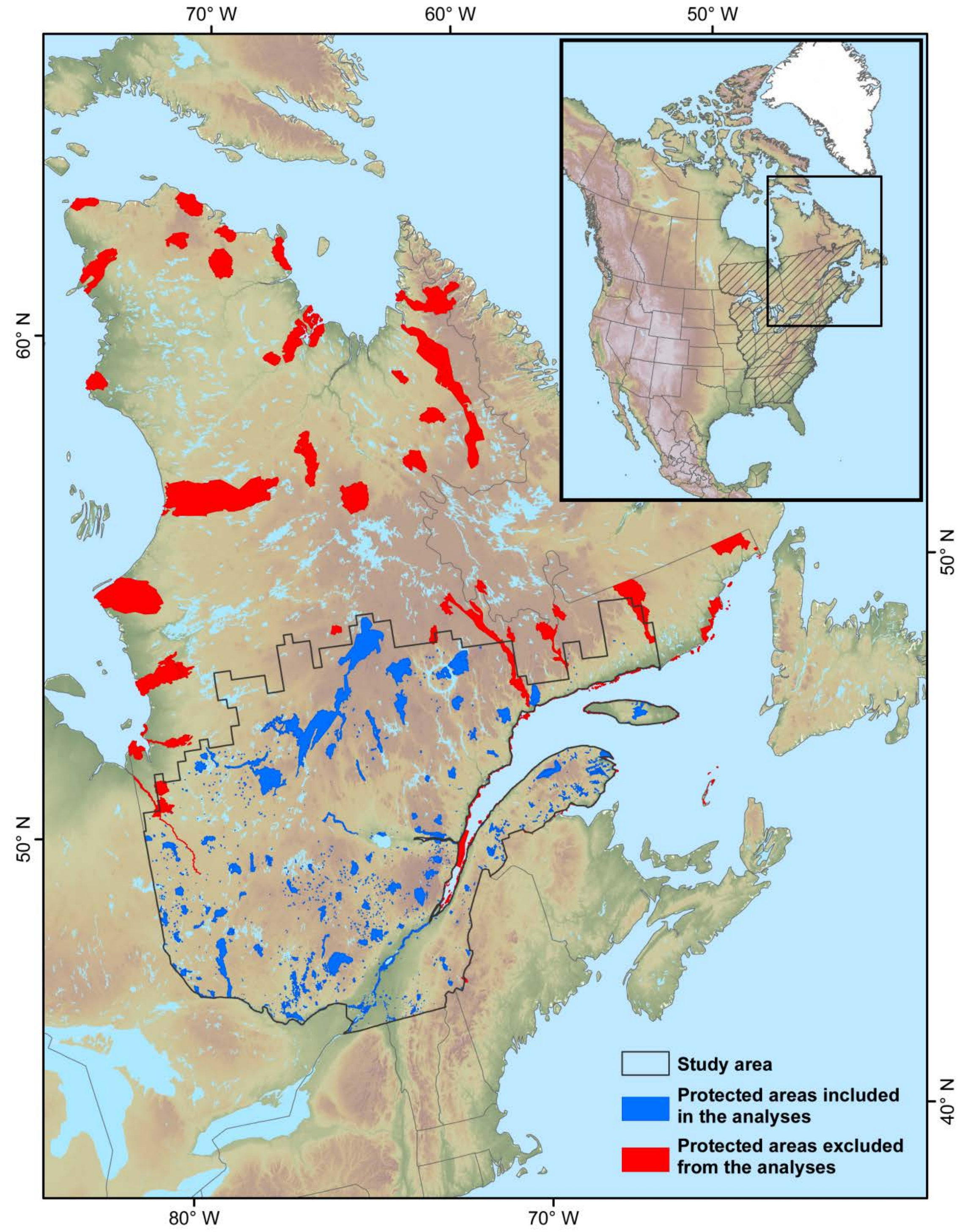

Supplementary Figure S4: Proportion of protected areas in each IUCN category according to size of protected areas in the study area. Protected areas that could be associated to multiple IUCN categories or for which classification has not yet been completed are identified as "others". The numbers of protected areas in each size class are indicated on top of bars.

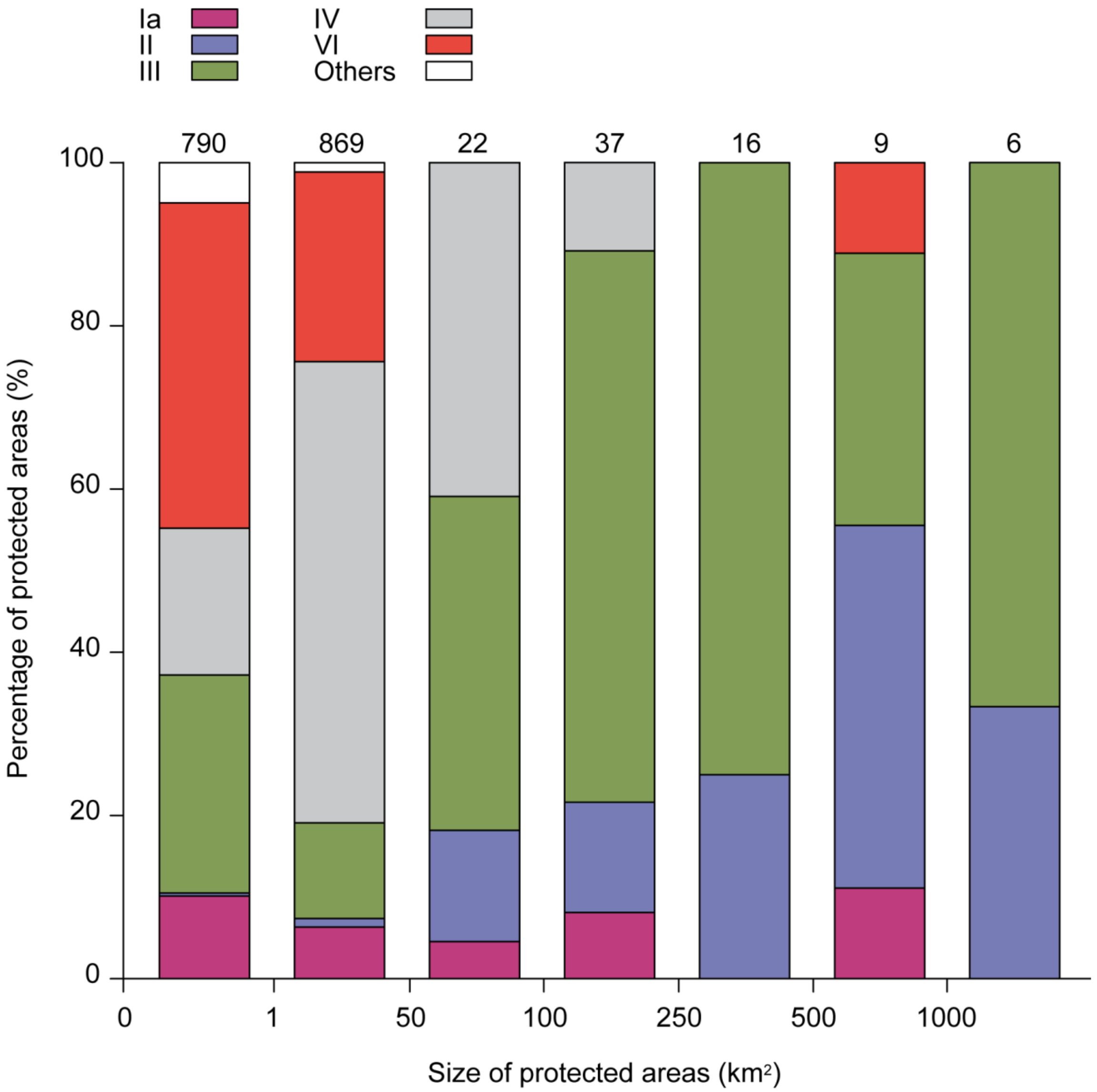

Supplementary Figure S5: Distribution of AUC values for the 158,920 models calibrated in this study. Calibrated models generally had a good predictive performance, with median AUC = 0.868 (sd = 0.069, n = 158,920). Note that models calibrated with random forests appear mostly in the right distribution peak of the histogram.

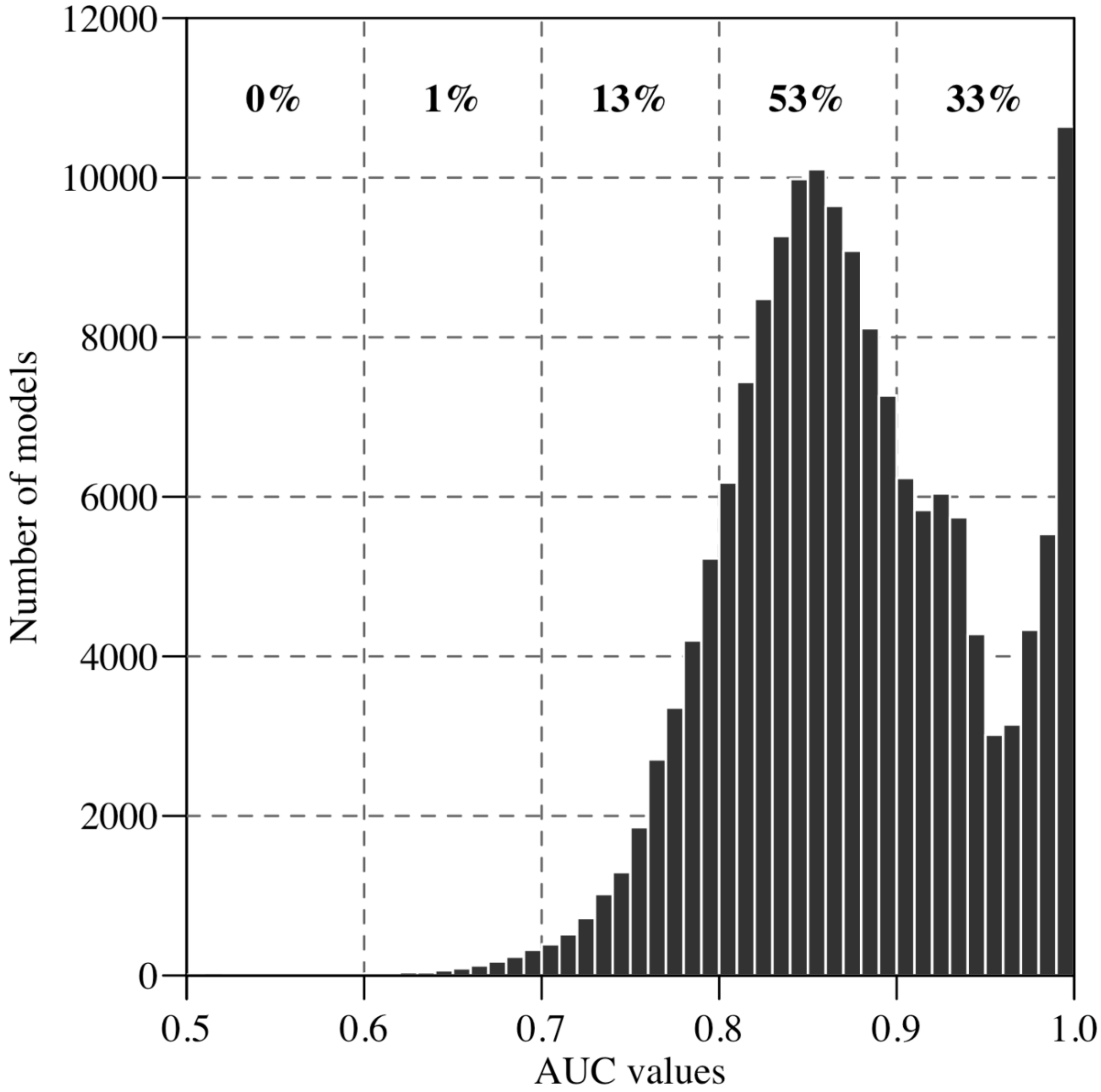

References

1. Thuiller, W., Lafourcade, B., Engler, R. & Araújo, M. B. BIOMOD - a platform for ensemble forecasting of species distributions. *Ecography* **32,** 369–373 (2009).
2. R Development Core Team. *R: A Language Environment for Statistical Computing.* (R Foundation for Statistical Computing, 2012).
3. Fielding, A. H. & Bell, J. F. A review of methods for the assessment of prediction errors in conservation presence/absence models. *Environmental conservation* **24,** 38–49 (1997).
4. Araújo, M. B., Pearson, R. G., Thuiller, W. & Erhard, M. Validation of species–climate impact models under climate change. *Global Change Biology* **11,** 1504–1513 (2005).
5. F. Dormann, C. *et al.* Methods to account for spatial autocorrelation in the analysis of species distributional data: a review. *Ecography* **30,** 609–628 (2007).
6. Bahn, V. & McGill, B. J. Testing the predictive performance of distribution models. *Oikos* **122,** 321–331 (2013).
7. Roberts, D. R. *et al.* Cross-validation strategies for data with temporal, spatial, hierarchical, or phylogenetic structure. *Ecography* **40,** 913–929 (2017).
8. Araújo, M. B. & New, M. Ensemble forecasting of species distributions. *Trends in Ecology & Evolution* **22,** 42–47 (2006).
9. Marmion, M., Parviainen, M., Luoto, M., Heikkinen, R. K. & Thuiller, W. Evaluation of consensus methods in predictive species distribution modelling. *Diversity and distributions* **15,** 59–69 (2009).

10. Liu, C., Berry, P. M., Dawson, T. P. & Pearson, R. G. Selecting thresholds of occurrence in the prediction of species distributions. *Ecography* **28,** 385–393 (2005).

11. Droege, S., Cyr, A. & Larivée, J. Checklists: An Under-Used Tool for the Inventory and Monitoring of Plants and Animals. *Conservation Biology* **12,** 1134–1138 (1998).

12. Iverson, L. R. & Prasad, A. M. Predicting abundance of 80 tree species following climate change in the eastern United States. *Ecological Monographs* **68,** 465–485 (1998).

13. Flora of North America Editorial Commitee, eds. *Flora of North America North of Mexico*. (Flora of North America Editorial Commitee, 1993).

14. Rousseau, C. *Géographie floristique du Québec-Labrador : distribution des principales espèces vasculaires*. (Québec : Presses de l'Université Laval, 1974).

15. Rehfeldt, G. E. *A spline model of climate for the Western United States*. (U.S. Department of Agriculture, Forest Service, Rocky Mountain Research Station, 2006).

16. Meehl, G. A. *et al.* The WCRP CMIP3 multimodel dataset: A new era in climate change research. *Bulletin of the American Meteorological Society* **88,** 1383–1394 (2007).

17. Nakicenovic, N. *et al. Special report on emissions scenarios: a special report of Working Group III of the Intergovernmental Panel on Climate Change*. (2000).

18. Caya, D. & Laprise, R. A semi-implicit semi-Lagrangian regional climate model: The Canadian RCM. *Monthly Weather Review* **127,** 341–362 (1999).

19. de Berg, M., Cheong, O., van Kreveld, M. & Overmars, M. *Computational geometry: algorithms and applications*. (Springer-Verlag Berlin Heidelberg, 2008).

20. Stocker, T. F. *et al.* Technical summary. in *Climate Change 2013: The Physical Science Basis. Contribution of Working Group I to the Fifth Assessment Report of the Intergovernmental Panel on Climate Change* 33–115 (Cambridge University Press, 2013).
21. Rogelj, J., Meinshausen, M. & Knutti, R. Global warming under old and new scenarios using IPCC climate sensitivity range estimates. *Nature Climate Change* **2,** 248–253 (2012).
22. Casajus, N. *et al.* An objective approach to select climate scenarios when projecting species distribution under climate change. *PLoS One* **11,** e0152495 (2016).
23. Iverson, L. R., Prasad, A. M., Matthews, S. N. & Peters, M. Estimating potential habitat for 134 eastern US tree species under six climate scenarios. *Forest Ecology and Management* **254,** 390–406 (2008).
24. *Guidelines for applying protected area management categories*. (IUCN, 2008).
25. Chambers, D., Périé, C., Casajus, N. & de Blois, S. Challenges in modelling the abundance of 105 tree species in eastern North America using climate, edaphic, and topographic variables. *Forest Ecology and Management* **291,** 20–29 (2013).
